# Podoplanin is a component of extracellular vesicles that reprograms cell-derived exosomal proteins and modulates lymphatic vessel formation

**DOI:** 10.18632/oncotarget.7445

**Published:** 2016-02-17

**Authors:** Patricia Carrasco-Ramírez, David W. Greening, Germán Andrés, Shashi K. Gopal, Ester Martín-Villar, Jaime Renart, Richard J. Simpson, Miguel Quintanilla

**Affiliations:** ^1^ Instituto de Investigaciones Biomédicas Alberto Sols, Consejo Superior de Investigaciones Científicas (CSIC) – Universidad Autónoma de Madrid (UAM), Madrid, Spain; ^2^ Department of Biochemistry and Genetics, La Trobe Institute for Molecular Science, La Trobe University, Melbourne, Victoria, Australia; ^3^ Electron Microscopy Unit, Centro de Biología Molecular Severo Ochoa, Consejo Superior de Investigaciones Científicas (CSIC) – Universidad Autónoma de Madrid (UAM), Madrid, Spain

**Keywords:** podoplanin, microvesicles, exosomes, tumor progression, lymphangiogenesis

## Abstract

Podoplanin (PDPN) is a transmembrane glycoprotein that plays crucial roles in embryonic development, the immune response, and malignant progression. Here, we report that cells ectopically or endogenously expressing PDPN release extracellular vesicles (EVs) that contain PDPN mRNA and protein. PDPN incorporates into membrane shed microvesicles (MVs) and endosomal-derived exosomes (EXOs), where it was found to colocalize with the canonical EV marker CD63 by immunoelectron microscopy. We have previously found that expression of PDPN in MDCK cells induces an epithelial-mesenchymal transition (EMT). Proteomic profiling of MDCK-PDPN cells compared to control cells shows that PDPN-induced EMT is associated with upregulation of oncogenic proteins and diminished expression of tumor suppressors. Proteomic analysis of exosomes reveals that MDCK-PDPN EXOs were enriched in protein cargos involved in cell adhesion, cytoskeletal remodeling, signal transduction and, importantly, intracellular trafficking and EV biogenesis. Indeed, expression of PDPN in MDCK cells stimulated both EXO and MV production, while knockdown of endogenous PDPN in human HN5 squamous carcinoma cells reduced EXO production and inhibited tumorigenesis. EXOs released from MDCK-PDPN and control cells both stimulated *in vitro* angiogenesis, but only EXOs containing PDPN were shown to promote lymphatic vessel formation. This effect was mediated by PDPN on the surface of EXOs, as demonstrated by a neutralizing specific monoclonal antibody. These results contribute to our understanding of PDPN-induced EMT in association to tumor progression, and suggest an important role for PDPN in EV biogenesis and/or release and for PDPN-EXOs in modulating lymphangiogenesis.

## INTRODUCTION

Podoplanin (PDPN, also known as PA2.26 antigen, OTS-8, Aggrus, D2-40 and T1α) is a small mucin-like transmembrane glycoprotein expressed in different tissues that plays a crucial role in development of the heart, lungs and lymphatic vascular system [1]. PDPN is upregulated in a variety of cancers, including squamous cell carcinomas (SCCs) and glioblastomas, generally associated with poor prognosis [1, 2]. Elevated expression of PDPN is often found at the leading edge of invasive tumor nests [3–8], and has been identified as a marker of a cell subpopulation with stem-like characteristics in SCC and glioblastoma cell lines [9–11]. PDPN is also expressed by inflammatory macrophages [12, 13] as well as cancer-associated fibroblasts of various malignancies [14–19]. Previously, we have reported that PDPN induces an epithelial-mesenchymal transition (EMT) in transformed keratinocytes and Madin-Darby canine kidney (MDCK) cells associated with increased migration/invasion and lymph node metastasis formation [20–22]. In addition, other studies have shown that PDPN can stimulate collective tumor cell migration/invasion in the absence of EMT [4, 23]. Of note, antibodies and lectins targeting PDPN halt the growth and dissemination of PDPN-expressing tumor cells [24–27].

PDPN is expressed at plasma membrane extensions, such as microvilli, filopodia and ruffles, where is linked to the actin cytoskeleton by binding ezrin and moesin, two members of the ERM (ezrin, radixin, moesin) protein family of cross-linkers between the plasma membrane and cytoskeleton [20, 22]. This interaction is crucial for PDPN-mediated activation of RhoA GTPase and its downstream kinase ROCK to promote EMT [22]. Recently, we and others have found that PDPN is a component of ventral membrane protrusions called invadopodia that mediate degradation of the extracellular matrix (ECM) by tumor cells [28, 29]. The presence of PDPN in these structures is related to invadopodia stability and promotion of efficient matrix proteolysis [29]. In addition, PDPN interacts with the standard isoform of the hyaluronan receptor CD44s on the surface of carcinoma cells [30]. This interaction seems to be crucial for tethering tumor cells to hyaluronan-rich ECMs [31] and for PDPN stimulation of directional migration [30]. PDPN also mediates cell adhesion through its interaction with the C-type lectin-like receptor 2 (CLEC-2), which is present on platelets and immune cells [1]. PDPN-mediated platelet aggregation via CLEC-2 interaction facilitates tumor cell growth and pulmonary metastasis [32, 33], whereas, in normal cells, this interaction is relevant for development of the lymphatic vasculature, lymphangiogenesis and the immune response [34–38]. In addition, PDPN is expressed in effector T cells and negatively regulates their survival in the target tissues, thus promoting tissue tolerance [39]. A fraction of PDPN is located in detergent-resistant membrane domains or lipid rafts [40, 41]. Importantly, the exclusion of PDPN from these platforms impairs promotion of EMT and cell migration [41].

Extracellular membrane vesicles (EVs) released by different types of cells are present in body fluids, such as blood, urine, semen and ascites, and serve as indicators in the diagnosis/prognosis of a variety of diseases [42–44]. EVs function as vehicles for intercellular communication, for transfer of proteins, lipids and RNA between cells (local or systemic), and have been involved in physiological and pathological processes, such as coagulation, inflammation, and tumor progression [42, 45–47]. These circulating vesicles are a heterogeneous population of membrane structures that for simplification have been classified into two major categories: exosomes (EXOs) and microvesicles (MVs) or ectosomas [48]. EXOs are vesicles of 40-150 nm in diameter of endosomal origin that are released after the fusion of multivesicular bodies (MVBs) with the plasma membrane [49]. MVs, on the other hand, are a more heterogeneous population of vesicles, with sizes ranging from 100 nm to ≥ 1 μm. MVs are directly shed from the cell surface in a process that involves outward budding and fission of the plasma membrane [50]. Tumor cells themselves and cells in the tumor microenvironment secrete MVs and EXOs, and increasing evidence suggests that both type of vesicles contribute to tumor progression by stimulating angiogenesis, facilitating evasion of the immune surveillance and promoting cell migration/invasion and metastasis [43–47].

In this article, we demonstrate that PDPN is incorporated into the membrane of both EXOs and MVs. Comparison of the protein profiles of MDCK cells following PDPN expression reveals extensive reprogramming in whole cells and EXOs associated with EMT and tumor progression. Furthermore, this study provides experimental evidence supporting a role for PDPN in stimulating EV production and for PDPN-expressing EXOs in modulating lymphangiogenesis.

## RESULTS

### PDPN is expressed in EVs released by different cell types

**Figure 1:**
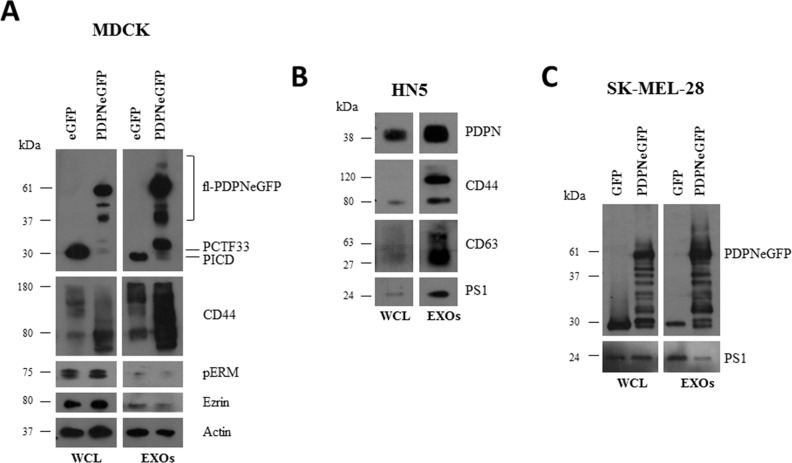
Western blot analysis of protein expression in whole cells and EVs isolated from different cell lines EVs were isolated as crude EXOs by sequential ultracentrifugation as described in Materials and methods. **A.** MDCK cells expressing either PDPNeGFP or eGFP. **B.** HN5 cells expressing endogenous PDPN. **C.** SK-MEL-28 cells expressing either PDPNeGFP or eGFP. WCL, whole cell lysate.

Ectopic expression of PDPN in MDCK cells (MDCK-PDPN) induces an EMT associated with downregulation of epithelial markers, such as E-cadherin and keratins, and induction of mesenchymal proteins; i.e. N-cadherin, allowing the conversion from an epithelial to a fibroblast-like phenotype [22] (Supplementary Figure S1A, S1B). In experiments aimed to ascertain whether PDPN is shed from the cell surface, we identified full-length PDPN in the conditioned medium (CM) from MDCK-PDPN cells (Supplementary Figure S2A). Similarly, human HN5 squamous carcinoma cells, which express endogenous PDPN, released full-length PDPN into the CM (Supplementary Figure S2B). Secreted PDPN was present in the pellet fraction following ultracentrifugation of the CM (Supplementary Figure S2A, S2B), revealing PDPN associated with EVs. All EV preparations that were isolated by standard procedures (crude EXO fractions; see Materials and methods) from different cell lines: HN5 cells and MDCK or human melanoma SK-MEL-28 cells expressing PDPN tagged with enhanced green fluorescent protein (MDCK-PDPNeGFP, SK-MEL-28-PDPNeGFP), contained PDPN when analyzed by Western blotting (Figure 1). The isolated crude EXO fractions were enriched for known exosomal proteins, such as CD63 and CD44 (Figure 1A, 1B). Ezrin and activated (phosphorylated) ERM proteins known to interact with both PDPN and CD44 [20, 22, 51] were also identified in EVs (Figure 1A). We have previously reported that PDPN undergoes a constitutive sequential proteolytic processing by an unknown metalloprotease followed by presenilin-1 (PS1)/γ-secretase resulting in a ~33 kDa C-terminal membrane-bound fragment (PCTF33) and a ~29 kDa cytosolic fragment containing the intracellular domain (PICD) [52]. Interestingly, both PCTF33 and PICD were identified together with the full-length protein (fl-PDPNeGFP) in the crude EXO fraction of MDCK-PDPNeGFP cells (Figure 1A), suggesting that PDPN cleavage may also occur in EVs, as it has been reported for CD44 and cadherin-17 [53, 54]. Indeed, the catalytic subunit of γ-secretase, PS1, is also incorporated into these vesicles (Figure 1B, 1C).

It has been reported that EVs contain select mRNAs that can be transferred to recipient cells and remain functional [55]. As the 3′ untranslated region of human PDPN mRNA contains a reported CTGCC core 25-nucleotide “zipcode” sequence (Supplementary Figure S3A) that can facilitate enrichment in EVs [56], we examined the presence of PDPN mRNA in EVs isolated from MDCK-PDPN and HN5 cells. Interestingly, a band of ~570 bp corresponding to PDPN mRNA was detected in the crude EXO fractions from these cells (Supplementary Figure S3B).

### Localization of PDPN in MVBs and EXOs by confocal immunofluorescence and electron microscopy analysis

**Figure 2:**
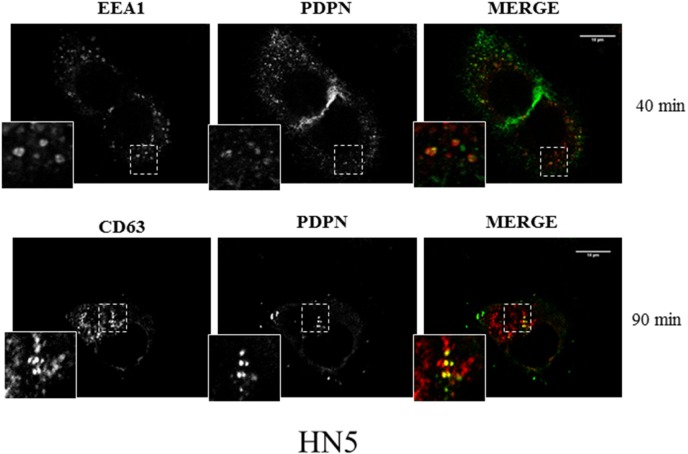
Confocal immunofluorescence reveals colocalization of PDPN with endosomal protein markers HN5 cells were labeled at 4°C with anti-PDPN Ab and, after washing, incubated at 37°C for 40 min to analyze the presence of PDPN in early endosomes labeled with anti-EEA1 (upper panel) and 90 min to analyze the presence of PDPN in late endosomes labeled with anti-CD63 (bottom panel). EEA1 and CD63 were detected using specific monoclonal Abs and AlexaFluor 546-conjugated goat anti-mouse IgG (red). PDPN was detected using AlexaFluor 488-conjugated goat anti-rabbit IgG (green). Scale bar, 10 μm.

**Figure 3:**
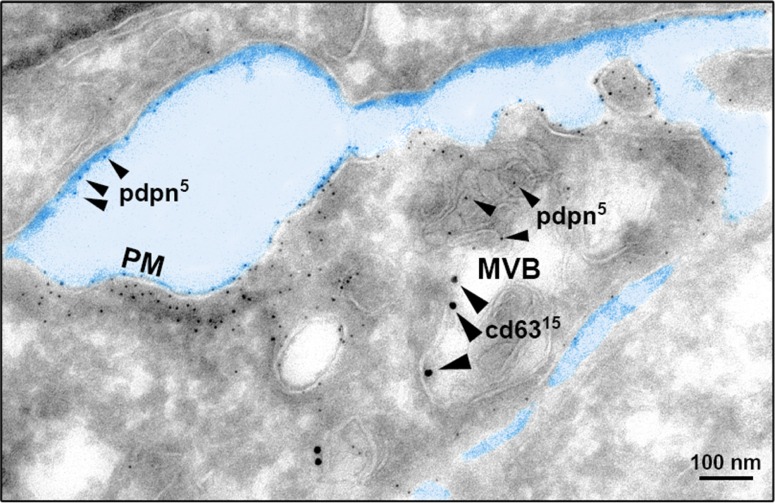
Cryoelectron microscopy reveals MVB colocalization of PDPN and CD63 PDPN was detected in SK-MEL-28-PDPNeGFP cells with a specific polyclonal Ab and protein A conjugated to 5-nm gold particles (small arrowheads), and CD63 with a specific monoclonal Ab and a goat anti-mouse conjugated to 15-nm gold particles (large arrowheads). Note the presence of PDPN in ILVs of MVBs, although most of PDPN labeling occurs at the plasma membrane (PM). Scale bar, 100 nm.

**Figure 4:**
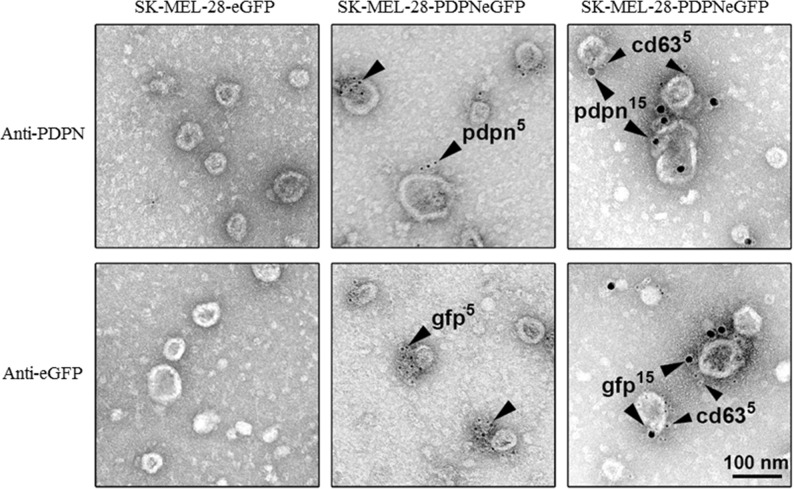
PDPN immunoelectron microscopy of EVs isolated from SK-MEL-28-PDPNeGFP cells PDPNeGFP was detected by two distinct rabbit polyclonal Abs directed either against the extracellular domain of PDPN (pdpn) or the intracellular tag GFP (gfp), and protein A conjugated to 5-nm gold particles (middle panels). The presence of CD63, an EXO marker, in EVs expressing PDPNeGFP was determined by double immunogold labeling using rabbit Abs against either PDPN or GFP and a specific monoclonal Ab to CD63. Signal was revealed by using an anti-rabbit Ab conjugated to 15-nm gold particles and an anti-mouse Ab conjugated to 5-nm gold particles (right panels). No signal was detected in control SK-MEL-28-eGFP cells. Scale bar, 100 nm.

In order to explore whether cell-surface PDPN is endocytosed through a clathrin-dependent pathway, HN5 cells were labeled at 4°C with an anti-PDPN antibody (Ab). Labeled cells were then incubated at 37°C, and the presence of PDPN in early and late endosomes was identified by confocal immunofluorescence co-localization with early endosomal antigen 1 (EEA1) and CD63, respectively (Figure 2). Furthermore, double immunogold labeling and electron microscopy analysis detected the presence of PDPN (5-nm particles) and CD63 (15-nm particles) in intraluminal vesicles of MVBs from thawed SK-MEL-28-PDPNeGFP cryosections (Figure 3). Most of PDPN labeling in SK-MEL-28-PDPNeGFP cryosections was found at the plasma membrane, suggesting that only a minor fraction of cell-surface PDPN is internalized or, alternatively, that PDPN is actively recycled from endosomes to the plasma membrane. PDPN and CD63 were also found to colocalize at the membrane of EVs released by these cells (crude EXO fraction) by electron microscopy analysis (Figure 4). Two different Abs recognizing PDPNeGFP, either directed against the ectodomain of PDPN (pdpn^5^ or pdpn^15^) or intracellular GFP (gfp^5^ or gfp^15^) were used. The EVs labeled for PDPN and CD63 were of 72±19 nm (n=20), suggesting that vesicles are EXOs. EXOs derived from MDCK-PDPN and MDCK-PDPNeGFP cells were also labeled for PDPN, while immunogold detection of CD63 was not observed due to the fact that the human-specific Ab did not recognize the canine protein epitope (Supplementary Figure S4).

### PDPN is identified in EV subtypes

**Figure 5:**
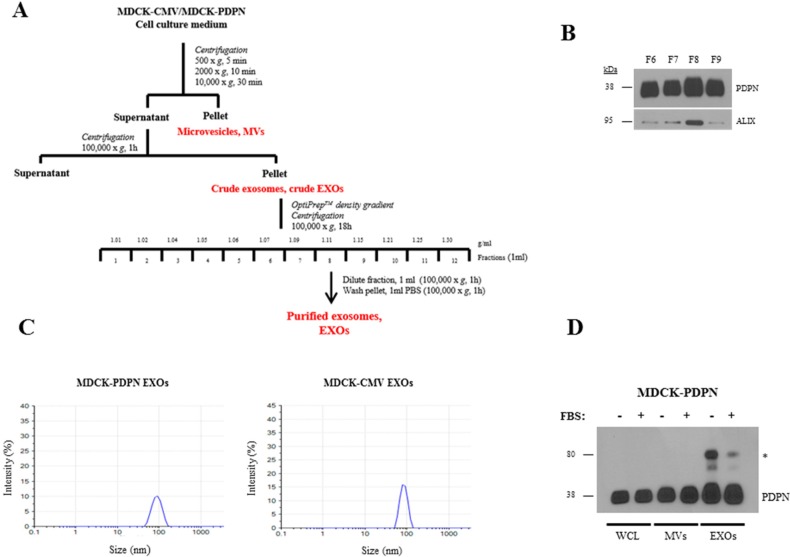
Purification and characterization of EXOs released from MDCK-CMV and MDCK-PDPN cells **A.** Experimental workflow for MV and EXO purification from MDCK-CMV and MDCK-PDPN cells. **B.** Western blot analysis of PDPN and Alix expression in fractions 6-9 from OptiPrep density-gradient performed with the crude EXO preparation from MDCK-PDPN cells. Note that significant enrichment of both Alix and PDPN were observed in fraction 8. **C.** Dynamic Light Scattering of EXOs from MDCK-PDPN and control MDCK-CMV cells purified by OptiPrep density-gradient fractionation. **D.** Western blot analysis of PDPN expression in MDCK-PDPN cell lysate (WCL) and corresponding MV and EXO fractions derived from the conditioned medium in the presence/absence of 10% FBS (EV-depleted). * indicates a band of ~80 kDa that corresponds to a PDPN non-covalent homodimer identified in normal and tumor tissues [41].

Since the EV fraction is a heterogeneous population mainly composed of endosomal derived EXOs and plasma membrane shed MVs [48], we asked whether PDPN is associated with only one or with both types of EVs. To this end, we isolated MVs and EXOs from MDCK-PDPN and MDCK-CMV control cells. EVs were isolated by differential centrifugation, and the crude EXO fraction obtained after ultracentrifugation, with purification using a density-based gradient fractionation, as outlined in Figure 5A. Western blot analysis of individual fractions revealed enrichment of EXOs (based on the exosomal marker Alix) and PDPN in fraction 8 (F8) with a buoyant density of 1.11 g/ml (Figure 5B). Dynamic light scattering analysis of EXO fractions from MDCK-CMV and MDCK-PDPN cells revealed a homogeneous distribution of ~ 85- and 90-nm mean particles, respectively (Figure 5C). Expression of PDPN in MVs and EXOs released by MDCK-PDPN cells in the absence or presence of 10% serum (EV-depleted) was compared by gel-based analysis of the same amount of total protein (10 μg) of EVs and cell lysates. Western blotting revealed that PDPN is present in MVs and EXOs, and that the presence of serum did not influence the incorporation of PDPN into either EV subtype (Figure 5D).

### PDPN regulates EV production

**Figure 6:**
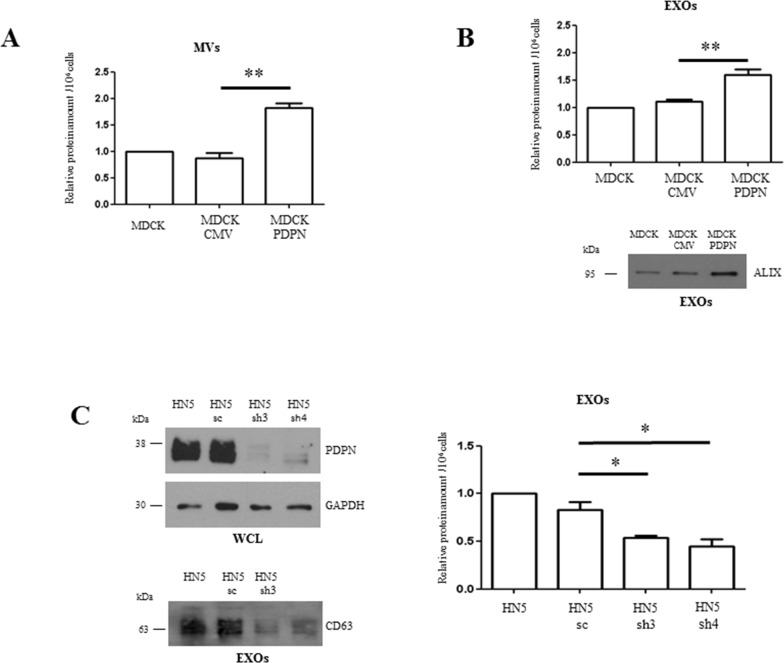
PDPN facilitates increased production and release of EV subtypes **A, B.** Quantification of MVs (A) and EXOs (B) released by MDCK, MDCK-CMV and MDCK-PDPN cells. MVs and crude EXOs were isolated from the same number (2×10^6^) of seeded cells, as indicated in the workflow presented in Figure 5A. After CM collection, cells were counted and used for normalization. EVs isolated from the CM were resuspended in the same volume (50 μl) of PBS, quantified and represented as a proportion per 10^6^ cells. In panel B, a Western blot of Alix expression, as a protein marker of EXOs, is presented. The same volume (20 μl) of the crude EXO fractions was loaded onto each lane. **C.** Quantification of EXOs released by HN5 in response to PDPN knockdown. Two specific shRNAs (sh3, sh4) and shRNA control (sc) were used to deplete endogenous PDPN from HN5 cells. In the upper panel, a Western blot showing PDPN downregulation in HN5 cells in which GAPDH was used as a control of protein loading is presented. Aliquots of total cell lysates containing equivalent amount of proteins (30 μg) were loaded. In the lower panel, the Western blot shows diminished expression of CD63 as a measure of EXOs released by PDPN-knockdown cells. The same volume (20 μl) of crude EXO fractions were loaded. Results are expressed as the mean of three independent experiments α s.e.m. **p < 0.01 (A, B); *p < 0.05 (C).

It is widely accepted that production of EVs is upregulated during tumor progression [47]. Since PDPN expression in tumor cells is associated with increased malignancy [1, 2, 20–23], we explored whether PDPN modulates EV biogenesis. To address this aim, we quantified protein content of MVs and crude EXOs isolated from the same number of MDCK, MDCK-CMV and MDCK-PDPN cells (Figure 5A). Parental MDCK and control MDCK-CMV cells produced similar amounts of MVs and EXOs, while in MDCK-PDPN cells the amount of these vesicles was significantly enhanced ~1.5-1.7-fold (Figure 6A, 6B, upper panels). An increase in the content of Alix was also observed in MDCK-PDPN with respect to parental and control cells (Figure 6B, bottom panel). MDCK and derived cell lines produced ~10-fold more EXOs than MVs. Typically, the amount of MVs per 10^6^ cells was 0.25 μg (MDCK, MDCK-CMV) to 0.4 μg (MDCK-PDPN), while crude EXO amounts were 2.8 μg (MDCK, MDCK-CMV) to 4.2 μg (MDCK-PDPN).

The amount of EXOs produced by human HN5 squamous carcinoma cells after PDPN knockdown by small hairpin RNA (shRNA) interference [30] was also quantified. Production of EXOs was reduced ~2-fold after downregulation of PDPN expression (>80%; see Figure 6C, left, upper panel), as measured by protein quantification (Figure 6C, right) and Western blot analysis of CD63 (Figure 6C, left, lower panel). Absolute values for EXOs were: 0.1-0.2 μg per 10^6^ HN5-sh cells in comparison to 0.2-0.4 μg per 10^6^ control cells. The amount of MVs produced by the HN5 cellular system was negligible. Moreover, the decreased production of EXOs by HN5-sh3 and HN5–sh4 cells with respect to control HN5-sc cells correlates with a drastic reduction of the tumorigenic potential of HN5 in nude mice. Whereas HN5-sc cells gave rise to tumors in all injection sites, the incidence of tumors induced by HN5-sh3 and HN5-sh4 cells decreased to 33% and 17%, respectively (Table 1). Taken together, these results indicate that PDPN stimulates EV biogenesis according to tumor progression.

**Table 1:** Tumorigenicity of the HN5-derived cell lines in nude mice

CELL LINE	NUMBER OF MICE WITH TUMORS	TUMORS / INJECTION SITES	LATENCIES (wks)^a^
HN5-sc	3/3	6/6	4-8
HN5-sh3	1/3	2/6	6-7
HN5-sh4	1/3	1/6	7

a The latency period is the time (weeks) needed for tumors to reach a size of 0.5 cm^2^

### Cellular proteome analysis of MDCK-CMV and MDCK-PDPN

**Figure 7:**
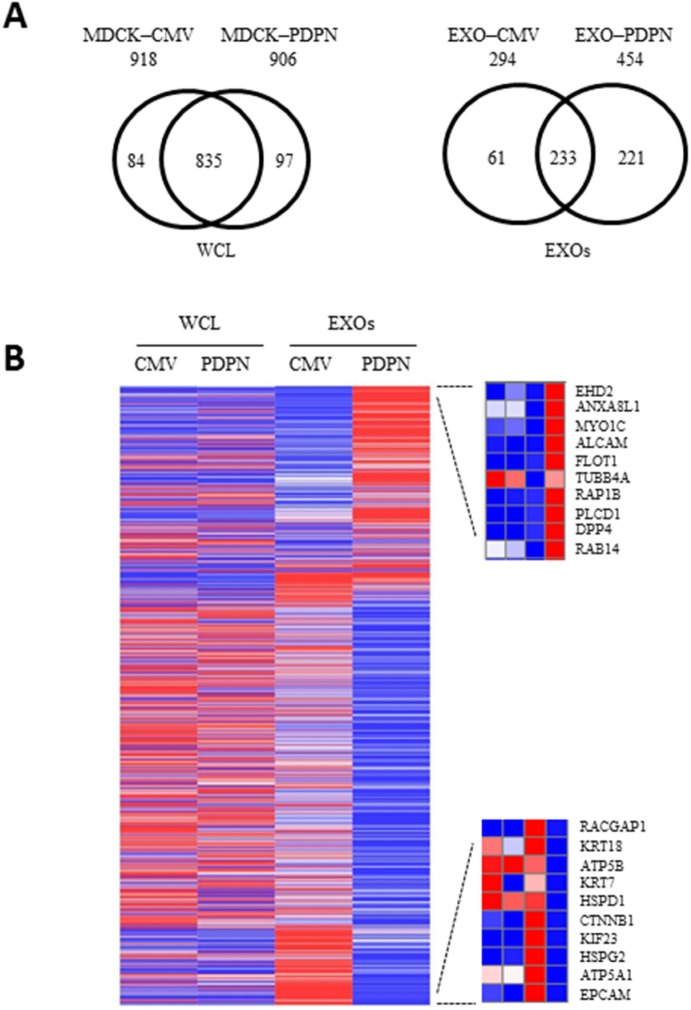
Proteomic profiling of MDCK cells and EXOs following PDPN overexpression **A.** Two-way Venn diagram of proteins commonly and uniquely identified in MDCK-CMV and MDCK-PDPN cells and EXOs. **B.** Heatmap representing proteins for which an increase (red) or decrease (blue) in protein expression was observed in MDCK-PDPN EXOs compared to control MDCK-CMV EXOs. For EXOs, differentially expressed upregulated (upper) and downregulated (bottom) proteins are shown.

The protein profiles of MDCK-CMV and MDCK-PDPN cell lysates were compared to reveal key insights into cellular changes as a consequence of PDPN expression. To investigate this, we cultured cells that were serum-starved for 24 h (to avoid serum-derived protein contamination in the CM). Serum-free conditions did not affect cell proliferation or viability (Supplementary Figure S5A, S5B). Using extensive and stringent informatics (protein FDR 1%, PEP 5%), the cellular proteomes retain a significant level of overlap with 835 proteins commonly detected for MDCK-CMV and MDCK-PDPN cells while 97 and 84 proteins were uniquely detected in MDCK-PDPN and MDCK-CMV cells, respectively (Figure 7A, left). A list of cellular proteins highly enriched in MDCK-PDPN in comparison to control MDCK-CMV cells is shown in Supplementary Table S1. These proteins are involved in actin dynamics and focal adhesion (PLS3, EHD2, EPS8, FERMT2), ECM remodeling (PLOD2, SERPINE1, CLIA2), intracellular trafficking and protein translocation (EHD2, KTN1, EPS8, SCFD1, PTPN1, SSR1), signal transduction (GNAS and CD109) and metabolism (HADHB, GLS, CYP51A1, PTPN1), and most of them have been found to promote tumorigenesis and/or metastasis (see Supplementary Information). Similarly, proteins highly downregulated in MDCK-PDPN cells with respect to control are listed in Supplementary Table S2. They comprise cell adhesion, cytoskeletal components and ECM remodeling proteins (KRT7, KRT14, TES, JUP, EPCAM, CTNNB1, TGM2, SERPINB5), as well as proteins involved in the control of intracellular vesicle and protein transport (ANXA6, AP1B1, LLGL2, RAB8A, COPG2 and TOMM40), signal transduction (CTNNB1 and SPTBN1), metabolism (PLA2G7, IDH, TSTA3, ACY1, PFAS), protein synthesis (EIF4A2 and EIF4G1), RNA processing (DDX47, ESRP1) and regulators of the ubiquitin pathway (ISG15 and TRIM28). Many of them seem to behave as tumor suppressors (see Supplementary Information).

### Proteome analysis of EXOs released by MDCK-CMV and MDCK-PDPN cells

The protein profiles of MDCK-CMV and MDCK-PDPN purified EXOs were compared to reveal key insights into cargo-specific changes as a consequence of PDPN expression. The protein profiles of EXOs revealed 233 proteins common for MDCK-PDPN and MDCK-CMV control cells, with EXOs from MDCK-PDPN cells enriched in specific proteins (Figure 7A, right). The top ten of these proteins are listed in the heat map of Figure 7B, right, upper panel, with a significant portion involved in vesicle trafficking and sorting (EHD2, ANXA8L1, MYO1C, FLOT1, RAP1B, RAB14). In fact, a large number of proteins enriched in MDCK-PDPN EXOs are implicated in the control of endocytosis and vesicle trafficking (Supplementary Table S3). These include members of the Rab, Ral an Rap subfamilies of small GTPases (RAB14, RAB1B, RAB13, RAB8B, RAB21, RAB35, RAB2A, RAB7A, RAB6A, RALA, RALB, RAP1B and RAP2B), annexins (ANXA8L1, ANXA4, ANXA7 and ANXA5), tetraspanins (CD151, CD82 and TSPAN9), components of endosomal sorting complex required for transport (ESCRT) machinery (TSG101 and IST1), members of soluble N-ethylmaleimide-sensitive fusion attachment protein (SNAP) receptors (SNARE) machinery (STX7 and STXB3), and proteins involved in lipid raft formation (FLOT1, FLOT2, ZDHHC5), among others. Interestingly, the expression of most of these proteins are not significantly increased in whole cells, with the exception of EHD2, CD82 and ANXA5 that were also upregulated in MDCK-PDPN cells (Supplementary Table S3).

Other proteins enriched in MDCK-PDPN EXOs are involved in cell adhesion: integrins and members of the immunoglobulin family, cytoskeletal remodeling: tubulins, myosin and kinesin molecular motors, actin-binding proteins (Supplementary Table S4), and components of signal transduction pathways, particularly those involved in semaphorin and ephrin pathways (Supplementary Table S5).

**Figure 8:**
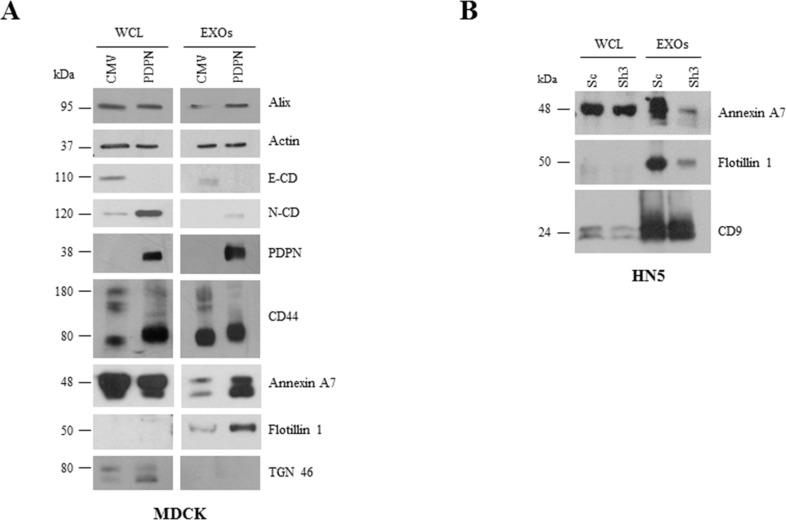
Comparative Western blot analysis of protein expression in cells and EXOs from MDCK and HN5 cell systems **A.** E-cadherin (E-CD) was downregulated while mesenchymal N-cadherin (N-CD) was upregulated in MDCK-PDPN cells and EXOs. Annexin A7 and flotillin 1 were upregulated in MDCK-PDPN EXOs. Both MDCK-PDPN and MDCK-CMV EXOs contained actin and CD44 and absence of the Golgi marker TGN46. **B.** Annexin A7 and flotillin 1 were downregulated in EXOs derived from PDPN knockdown HN5-sh3 cells while CD9 levels remained unchanged.

A number of proteins were highly downregulated in MDCK-PDPN EXOs with respect to EXOs from control cells. These include mitochondrial ATP synthases (ATP5A1, ATP5B) and chaperonin (HSPD1), the component of ECM perlecan (HSPG2), the Rac GTPase activating protein RACGAP1, the kinesin-like protein KIF23/MKLP1 as well as cytoskeletal and cell adhesion proteins (EPCAM, CTNNB1, KRT7, KRT18) (Figure 7B, right, lower panel). Indeed, EXOs from MDCK-CMV and MDCK-PDPN cells showed the pattern of EMT changes seen in whole cells with downregulation of epithelial markers, such as components of cell-cell junctions: epthelial cell adhesion molecule (EPCAM), E-cadherin (CDH1), catenins (CTNNA1, CTNNB1), claudin 4 (CLDN4), desmoglein 3 (DSG3), and cytoskeletal keratins (KRT7, KRT19, KRT18, KRT8) (Supplementary Table S6), and upregulation of mesenchymal proteins, such as N-cadherin (Figure 8A). The only exception was keratin 14 (KRT14) that was enriched by ~5 fold in MDCK-PDPN EXOs while downregulated by ~6 fold in whole cells (Supplementary Table S4). The significance of this observation is presently unknown.

EXO preparations from MDCK-CMV and MDCK-PDPN cells contained actin along with canonical markers Alix and CD44, and were negative for the Golgi marker TGN46 (Figure 8A), reinforcing the use of highly purified EXO populations. The increase in annexin A7 and flotillin 1 proteins in MDCK-PDPN EXOs found by proteomic analysis (Supplementary Table S3) was validated by Western blotting (Figure 8A). Likewise, the levels of these proteins decreased in EXOs released from PDPN-downregulated HN5-sh3 cells compared to control HN5-sc EXOs (Figure 8B). The levels of Alix and CD9 remained unchanged in EXOs derived from MDCK and HN5 cell systems, respectively (Figure 8A, 8B).

### Exosomes released from MDCK-PDPN cells stimulate lymphatic vessel formation

**Figure 9:**
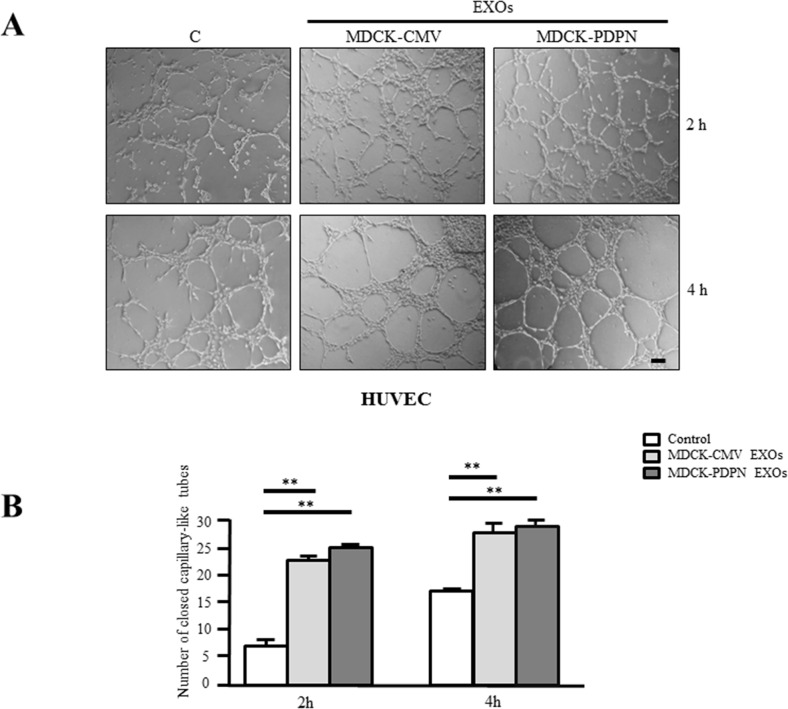
MDCK-PDPN and MDCK-CMV-released EXOs stimulate *in vitro* angiogenesis Representative micrographs **A.** and quantitative evaluation **B.** of the formation of closed capillary-like structures by HUVECs seeded on Matrigel-coated wells untreated (Control) or treated with MDCK-CMV and MDCK-PDPN crude EXOs (40 μg/ml). Data are expressed as the number of closed tubes per field. Bar, 150 μm. **p < 0.01. A representative experiment out of three is presented.

**Figure 10:**
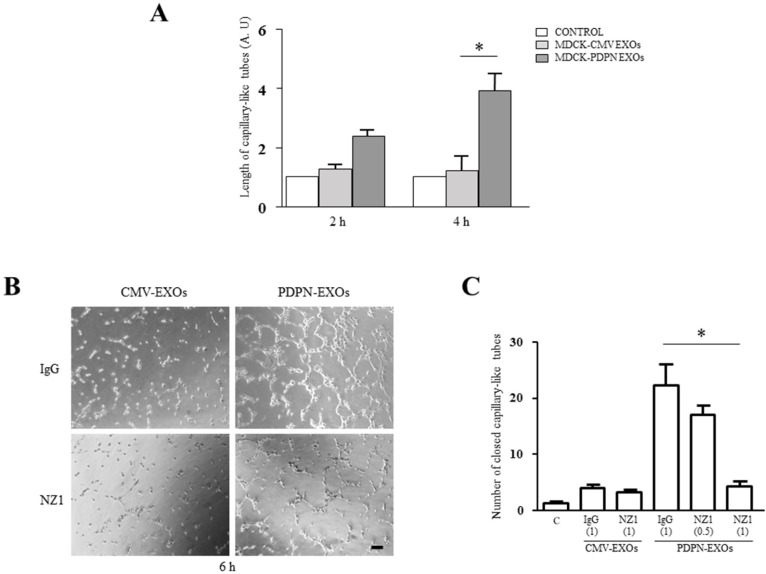
MDCK-PDPN-released EXOs stimulate *in vitro* lymphangiogenesis **A.** Quantitative evaluation of the length of tubes per field formed by HLECs seeded on Matrigel-coated wells untreated (Control) or treated with MDCK-CMV and MDCK-PDPN crude EXOs (40 μg/ml) for 2 h and 4 h. A representative experiment out of two is presented. **B, C.** Representative micrographs (B) and quantitative evaluation of the number of closed capillary-like structures per field (C) formed by HLECs seeded on Matrigel-coated wells untreated (Control) or treated with MDCK-CMV and MDCK-PDPN crude EXOs (40 μg/ml) for 6 h. EXOs were preincubated with mAb NZ1 (0.5 μg/ml and 1 μg/ml) recognizing the extracellular domain of PDPN or control IgG (1 μg/ml), as indicated, for 1h at 4°C. Bar, 100 μm. *p < 0.05. A representative experiment out of two is presented.

Next, we evaluated the effect of EXOs derived from MDCK-CMV and MDCK-PDPN cells on *in vitro* angiogenesis and lymphangiogenesis by measuring the ability of primary human umbilical vein endothelial cells (HUVEC) and human dermal lymphatic endothelial cells (HLECs) to organize into capillary-like structures on Matrigel. Both MDCK-CMV and MDCK-PDPN EXOs were able to stimulate the formation of HUVEC capillary-like tubes at the same extent (Figure 9A, 9B). However, only EXOs from MDCK-PDPN cells were able to promote *in vitro* lymphangiogenesis (Figure 10A–10C). PDPN-EXOs significantly stimulated both the length of tubes (Figure 10A) and the number of closed capillary-like structures (Figure 10B, 10C) formed by HLECs. The formation of lymphatic vessels was effectively inhibited by the anti-PDPN specific monoclonal antibody NZ1 in a dose-dependent manner, but not by control IgG (Figure 10B, 10C), suggesting that modulation of *in vitro* lymphangiogenesis by PDPN-EXOs is mediated by PDPN.

## DISCUSSION

We demonstrate that PDPN is secreted into the extracellular milieu as a component of different types of EVs: MVs and EXOs. Accordingly, PDPN should be added to the large list of lipid raft-associated proteins [40, 41] present in EVs [44]. EVs released by PDPN-expressing cells not only transport the protein but also the mRNA, reinforcing the possibility of PDPN transfer to target cells.

### PDPN-induced EMT is associated with malignant progression

PDPN expression in MDCK cells promotes an EMT linked to increased cell migration and invasiveness [22]. Likewise, by comparing the proteome profiles of MDCK-CMV and MDCK-PDPN cells, we show in this article that PDPN-induced EMT is associated with a reprogramming of proteins that favors tumor growth, invasiveness and metastasis. Thus, many proteins highly upregulated in MDCK-PDPN cells are pro-oncogenic and found to be overexpressed in different types of cancers, whereas most cellular proteins highly downregulated in MDCK-PDPN cells behave as tumor suppressors (Supplementary Tables S1 and S2 and Supplementary Information). These data provide an explanation for the acquisition of tumorigenic and metastatic capabilities observed in premalignant keratinocytes upon expression of PDPN [20, 21], and for the drastic reduction of tumorigenicity found in PDPN knocked-down HN5 carcinoma cells (Table 1).

### PDPN modulates reprogramming of exosomal protein cargos

Our data reveal both quantitative and qualitative protein enrichment in EXOs containing PDPN with respect to control EXOs. The increase in protein content in MDCK-PDPN EXOs could be related to malignant progression, as EXOs from highly malignant cells contain higher amounts of proteins than EXOs from non-malignant cells [57]. Interestingly, most proteins enriched in MDCK-PDPN EXOs were not overexpressed in whole MDCK-PDPN cells relative to MDCK-CMV control cells. These include cell-ECM and cell-cell adhesion receptors, cytoskeletal remodeling proteins, and components of signal transduction pathways, particularly those belonging to the semaphorin and ephrin pathways (Supplementary Tables S4 and S5). Semaphorins and ephrins besides their roles in axon guidance and development of the nervous system have been involved in angiogenesis, tumor invasion and metastasis, as well as in the recruitment and activation of tumor-associated immune cells [58–60].

Notably, MDCK-PDPN EXOs were enriched in proteins implicated in the regulation of intracellular vesicle trafficking (Supplementary Table S3). In fact, the most abundant protein present in MDCK-PDPN EXOs relative to control is EHD2, which besides regulating cell adhesion and motility is clearly implicated in the control of endocytic transport, although its precise function is still unknown [61]. Other proteins enriched in MDCK-PDPN EXOs are involved in EXO biogenesis and release; i.e., components of the ESCRT and SNARE machineries [44], or coordination of intracellular vesicle transport; i.e., tetraspanins, annexins and small Rab, Ral and Rap GTPases. Besides their role on membrane trafficking, tetraspanins participate in multitude of biological processes, including signal transduction, cell adhesion and motility and tumor cell invasion [62]. Interestingly, PDPN has been found to interact with the ubiquitous tetraspanin CD9 in tetraspanin-enriched membrane microdomains [63]. CD9 is present in both EXOs and MVs [64, 65], and whether this interaction is instrumental for PDPN segregation with these vesicles remains to be investigated. Among the RAB small GTPases enriched in MDCK-PDPN EXOs, RAB35 has been involved in exosome release mainly associated to early sorting endosomes [66]. Also, RAB14 and RAB21 are markers of early endosomes [67], whereas RAB7A located in late endosomes and lysosomes is involved in the secretion of Alix-containing EXOs [68]; another lysosomal protein enriched in MDCK-PDPN EXOs is LAMP1. These observations suggest that MDCK-PDPN EXOs derived from different subpopulations of MVBs that fuse with the plasma membrane, as it has been generally observed for EXOs secreted by different cell types [44]. Cytoskeletal proteins that are involved in the mobilization, docking and fusion of MVBs with the cell surface, such as tubulins and associated molecular motors, kinesins and myosins [43] are also enriched in MDCK-PDPN EXOs (Supplementary Table S3).

The proteomic profile of EXOs derived from MDCK-PDPN cells has similarities with other EXO proteomes linked to EMT [69, 70]. In those cases, enrichment of proteins controlling vesicle-mediated trafficking was observed in EXOs derived from mesenchymal-like cells, which might result from changes on membrane fluidity and dynamics imposed by EMT [71]. In particular, comparison of the EXO protein profiles of MDCK-PDPN and MDCK-H-RAS cells reveals enrichment of 21 common proteins, including EHD2, ANXA4, ANXA5, CD151, TSPAN9, RAB21, RALA, ITGA6 and ITGAV among others. However, there are also important differences; i.e., increased expression of metalloproteases MMP1 and MMP14 is unique for EXOs from RAS-transformed MDCK cells [70], while EXOs from MDCK-PDPN but not MDCK-H-RAS cells are enriched in components of the ephrin and semaphorin pathways, indicating a high degree of specificity in protein cargos incorporated into either EXO type despite EMT.

### A novel function for PDPN as a modulator of EV biogenesis

The enhanced malignant characteristics of MDCK-PDPN with respect to control cells and the abundance of proteins implicated in vesicle biogenesis and secretion in MDCK-PDPN EXOs suggested a role for PDPN in promoting EV formation. A quantitative comparison of EVs isolated from the same number of control MDCK-CMV and MDCK-PDPN cells confirmed that this was the case. EV production (both EXOs and MVs) was significantly increased in MDCK-PDPN cells (Figure 6A, 6B). It can be argued, however, that this increase in EV formation was mostly due to EMT rather than to a direct effect of PDPN. Consequently, we performed a loss-of-function experiment by knockdown PDPN in HN5 SCC cells. PDPN silencing did not allow a major change in HN5 morphology, although these cells exhibited decreased spreading [30]. PDPN-downregulated cells exhibited reduced tumorigenicity and secreted ~2-fold less EXOs than control and parental cells (Figure 6C). Taken together, these results suggest a regulatory role for PDPN on EV formation and/or secretion. Since PDPN is anchored to the actin cytoskeleton by ERM proteins and induces a major rearrangement of the actin cytoskeleton by activating RhoA and RhoC small GTPases [22, 29], its role on EV biogenesis may be exerted on processes dependent on actin-myosin dynamics, such as endocytosis, membrane fusion and exocytosis [43, 72]. In this respect, it is worth mentioning that a synergistic interaction between invadopodia and EXO secretion in cancer cells by which inhibition of invadopodia formation greatly reduces EXO release into the conditioned medium has been reported [73]. We have found that silencing of PDPN in HN5 cells impairs invadopodia stability and function [29], which may also contribute to reduced EXO production observed in PDPN-downregulated cells.

### A role for PDPN-expressing EXOs as modulators of lymphatic vessel formation

In this report, we present preliminary evidence supporting a role for PDPN-EXOs in modulating lymphangiogenesis. Both PDPN-expressing and non-expressing EXOs were able to stimulate blood vessel angiogenesis (Figure 9), a feature shared by EVs released from different types of tumor cells [42, 43, 47]. However, only PDPN-EXOs, but not control EXOs, were able to stimulate the formation of capillary-like structures of HLECs embedded in Matrigel (Figure 10). This stimulatory effect was abolished by targeting PDPN on the membrane of EXOs with a specific monoclonal antibody, indicating that is mediated by PDPN. PDPN is expressed in lymphatic endothelial cells [20, 74], and previous reports have found a crucial role for this glycoprotein in capillary morphogenesis and polarized migration of lymphatic endothelial cells via regulation of Rho family GTPase activities [75, 76]. Interestingly, soluble PDPN (a recombinant fusion protein of the extracellular domain of PDPN and Fc region of IgG) was shown to inhibit *in vitro* and *in vivo* lymphangiogenesis [77]. It remains to be established whether the effect of PDPN-EXOs involves the binding of PDPN to lymphatic chemokines, such as CCL21 [78], the interaction with a cell-surface receptor or the internalization by lymphatic endothelial cells. This feature might be relevant for malignant progression as tumor cells releasing PDPN-EXOs may increase lymphangiogenesis around primary tumors and in the lymph nodes, thus contributing to the formation of the lymphvascular niche, and favor the spread of tumor cells to distant organs [79]. Experiments aimed to elucidate the effect of PDPN-EXOs on tumor lymphangiogenesis and metastasis are currently in progress.

## MATERIALS AND METHODS

### Cell culture, PDPN transfection, and RNA interference

MDCK cell transfectants stably expressing either untagged PDPN or PDPNeGFP have been previously described [3, 22]. SK-MEL-28-PDPNeGFP cells were obtained by infecting human melanoma SK-MEL-28 cells with a lentiviral vector containing PDPNeGFP [29]. Short-hairpin RNAs (shRNAs) targeting human PDPN mRNA cloned into pLKO.1 vector and HN5 PDPNshRNA cell transfectants have been described elsewhere [30]. HN5 and SK-MEL-28 cell lines identities were confirmed by short tandem repeat (STR) profiling and comparison with ATCC STR database.

Cells were routinely cultured in Dulbecco's modified Eagle's medium (DMEM) supplemented with 1% penicillin/streptomycin and 10% fetal bovine serum (FBS), at 37°C, in a humidified 5% CO_2_ atmosphere.

Cell proliferation and viability assays were performed by reduction of 3-(4,5-dimethylthiazol-2-yl)-2,5-diphenyltetrazolium bromide (MTT) and trypan blue dye-exclusion, as previously described [80].

### RT-PCR

Reverse transcription analysis for PDPN in EVs isolated from HN5 and MDCK-PDPN cells was performed as previously described [22, 30]. Specific primers were 5′-GTGCTGGAATTCCCCGATGTGG-3′ and 5′-TCAGGTACCCTGGGCGAGTACCTTCC-3′.

### Internalization and confocal immunofluorescence analysis

Internalization studies were carried out as described [81]. Briefly, cells were incubated in serum-free medium at 37°C for 30 min to allow digestion of serum-derived ligands. After washing twice with cold Hank's balanced salt solution supplemented with 20 mM Hepes, pH 7.2, and 2% BSA, cells were incubated with an anti-PDPN polyclonal Ab directed against the extracellular domain [3] at 4°C for 1h. After washing, cells labeled with anti-PDPN Ab on their surface were put at 37°C to allow endocytosis, and fixed (3.7% formaldehyde in PBS) and permeabilized (0.05% Triton X-100) at different times. Fluorescence detection of EEA1 and CD63 was performed with specific monoclonal Abs (1:200 dilution) from BD Bioscience and Immunostep, respectively, and AlexaFluor 546-conjugated goat anti-mouse IgG, as secondary Ab. Internalized PDPN was detected using AlexaFluor 488-conjugated goat anti-rabbit IgG (Life Technologies). Images were taken in a Zeiss LSM710 confocal laser-scanning microscope (x63 oil objective).

### EV isolation and purification

Isolation and purification of EVs was performed as previously described [70, 82]. Briefly, cells were grown to 70% confluence, washed with serum-free DMEM medium, and cultured in this medium for 24 h. Conditioned medium (CM) was harvested and centrifuged twice (480 × *g* for 5 min, 2000 × *g* for 10 min) to sediment floating cells and remove cell debris. CM was centrifuged at 10,000 × *g* for 30 min to isolate shed MVs, and the resultant supernatant was further centrifuged at 100,000 × *g* for 1 h at 4°C to sediment the crude EXO fraction.

Crude EXOs were further purified using an OptiPrep™ density gradient as described [83]. The gradient was formed by adding 3 ml each of 40%, 20%, 10% and 2.5 ml of 5% of iodixanol solution (Axis-Shield PoC, Norway) to a 14 × 89 mm polyallomer tube (Beckman Coulter). Crude EXOs were resuspended in 500 μl of PBS, loaded onto the top of the gradient, and centrifugation performed at 100.000 × *g* for 18 h at 4°C. Twelve individual 1 ml fractions were collected and each one was centrifuged at 100.000 × *g* for 1 h at 4°C. The supernatant were discarded and pellets washed with 1 ml PBS at 100.000 × *g* for 1 h at 4°C. After centrifugation, EXOs were resuspended in 50 μl of PBS. To determine the density of each fraction, a control OptiPrep™ gradient containing 1 ml of 0.25 M sucrose/10 mM Tris, pH 7.5 instead of sample was layered onto a second gradient and run in parallel. Fractions were collected as described, serially diluted 1:10,000 with water, and the concentration of iodixanol determined by measuring the absorbance at 244 nm using a molar extinction coefficient of 320 L g^−1^cm^−1^.

### Electron microscopy and immunogold labeling

For electron microscopy analysis, the crude EXO fraction isolated from the cell lines were deposited on Formvar carbon-coated grids and immunolabeled with rabbit anti-PDPN Ab [3], at 1:250 dilution, or anti-GFP Ab (A-6455, Life Technologies; 1:500 dilution) and protein A conjugated to either 5-nm or 15-nm nanoparticles (Cell Microscopy Center, Utrecht University, The Netherlands). CD63 was labeled with mouse anti-CD63 monoclonal Ab (1:100, clone H5C6, Developmental Studies Hybridoma Bank, The University of Iowa) followed by goat anti-mouse conjugated to 5-nm gold particles as described elsewhere [84]. For anti-GFP detection, EXOs were permeabilized with 0.1% saponin for 5 min before immunogold labeling. Samples were negatively stained with 2% uranyl acetate before visualization.

For immunogold labeling of cell cryosections, cells grown on 100-mm dishes were fixed in 2% paraformaldehyde and 0.2% glutaraldehyde in PHEM buffer (60 mM Pipes, 25 mM Hepes, 10 mM EGTA, 2 mM MgCl_2_) at pH 6.9 for 2 h, at room temperature, and kept in 1% paraformaldehyde in PHEM buffer at 4°C. Subsequently, cells were scraped and embedded in 10% (w/v) gelatin, cryoprotected for 16 h at 4°C with 2.3 M sucrose and frozen by immersion in liquid nitrogen. Samples were sectioned on an EM FCS cryo-ultramicrotome (Ultracut UCT, Leica) at −120°C. For immunogold labeling, thawed 90-nm-thick cryosections were incubated with rabbit anti-PDPN Ab (1:250) and mouse anti-CD63 monoclonal Ab followed by goat anti-mouse conjugated to 15-nm gold particles and goat anti-rabbit conjugated to 5-nm gold particles (Aurion, Wageningen, The Netherlands). Sections were stained with a mix of 1.8% methylcellulose and 0.4% uranyl acetate and visualized with a JEOL JEM 1010 (Tokyo, Japan) electron microscope at 80 kV. Images were recorded with a 4k × 4k CMOS F416 camera from TVIPS (Gauting, Germany).

### Dynamic light scattering

Dynamic light scattering was performed with a Zetasizer nanoseries instrument (Malvern Instruments Ltd., UK), employing a 20 mW helium/neon laser, 633 nm. All experiments were performed at 20 μg/ml in filtered PBS, in triplicate. Samples were studied at a constant temperature of 25°C. Light scattering from the sample was detected by a photomultiplier tube placed at 90° to the incident laser beam. EXO size data refers to the scattering intensity distribution (z-average) with standard deviation provided (Malvern software).

### Protein quantification and immunoblotting

Cells were washed (ice cold PBS) and lysed on ice with SDS sample buffer (4% SDS, 20% glycerol, 0.01% bromophenol blue, 0.125 M Tris-HCl, pH 6.8. Lysates were subjected to ultracentrifugation for 30 min (336,000 × *g*, TLA-100 rotor, Beckman Coulter), and soluble supernatants retained for downstream use, or frozen at −80°C. Protein quantification was performed as previously described [70].

For immunoblotting, membranes were probed with primary antibodies for 1 h in TTBS (50 mM Tris, 150 mM NaCl, 0.05% Tween 20) followed by appropriate secondary Abs coupled to horseradish peroxidase. Primary Abs for phospho-ERM (1:1000), Alix (1:1000) and N-cadherin (1:1000) and TGN46 (1:1000) were from Cell Signaling; for ezrin (1:5000) and β-actin (1:10000) from Sigma Aldrich; for CD63 (1:1000) and GFP (1:5000) from Calbiochem; for PS1 (1:1000) from Abcam; for TSG101 (1:1000) and flotillin 1 (1:1000) from BD Transduction Laboratories; for CD9 (1:1000) and annexin A7 (N-19, 1:1000) from Santa Cruz Biotechnology; for GAPDH (1:1000) from Merck Millipore; for PDPN from Acris Antibodies (NZ1, 1:1000). For E-cadherin and CD44 detection, the monoclonal Abs ECCD2 and HP2/9 (a generous gift of Dr. F. Sánchez-Madrid), respectively, were used at 1:1000 dilution. Peroxidase activity was developed using an enhanced chemiluminiscence kit as indicated by the manufacturer (Pierce).

### Proteomic analysis

Proteomic analyses were performed for cell lysates and purified EXOs (10 μg protein) as previously described [85] with modifications. Proteomic experiments were performed in biological duplicates and technical replicate. Samples were lysed in SDS sample buffer, electrophoresed by SDS-PAGE and visualized by Imperial™ Protein Stain (Thermo Fisher Scientific). Individual samples were excised (single gel bands and multi-band excision) and destained (50 mM ammonium bicarbonate/acetonitrile), reduced (10 mM DTT, Calbiochem, for 30 min), alkylated (50 mM iodoacetic acid, Fluka, for 30 min) and trypsinized (0.2 μg trypsin, Promega Sequencing Grade, for 16 h at 37°C), as described [85].

For all samples, peptides were desalted using reverse-phase C18 StageTips [86], and eluted in 85% acetonitrile (ACN) in 0.5% formic acid (FA). Peptides were lyophilised in a SpeedVac and acidified with buffer containing 0.1% FA, 2% ACN. A nanoflow UPLC instrument (Ultimate 3000 RSLCnano, Thermo Fisher Scientific) was coupled on-line to an Orbitrap Elite mass spectrometer (Thermo Fisher Scientific) with a nanoelectrospray ion source (Thermo Fisher Scientific). For cellular and EXOs samples, ~ 3 μg peptides were loaded (Acclaim PepMap100 C18 5μm 100Å, Thermo Fisher Scientific) and separated (Vydac MS C18-RP column, 25 cm, 75 μm inner diameter, 3 μm 300Å, Grace, Hesperia, CA) with a 120-min linear gradient from 0-100% phase B (0.1% FA in 80% ACN) at a flow rate of 250 nL/min. Details of the mass spectrometer operation are described previously [87].

### Database searching and protein identification

Raw data were processed using Proteome Discoverer (v1.4.0.288, Thermo Fischer Scientific) enlisting a Human-Canine-Bovine-only (UniProt #195,909 entries) sequence databases (Apr-2015). Peptide lists were generated from a tryptic digestion with up to two missed cleavages, carbamidomethylation of cysteines as fixed modifications, and oxidation of methionines and protein N-terminal acetylation as variable modifications. Precursor mass tolerance was 10 ppm, product ions were searched at 0.6 Da tolerances, min peptide length defined at 6, maximum peptide length 144, and max delta CN 0.05. Peptide spectral matches (PSM) were validated using Percolator based on q-values at a 1% false discovery rate (FDR) [88, 89]. With Proteome Discoverer, peptide identifications were grouped into proteins according to the law of parsimony and filtered to 1% FDR [90]. Scaffold (Proteome Software Inc., Portland, OR, v 4.3.4) was employed to validate MS/MS-based peptide and protein identifications from database searching. Initial peptide identifications were accepted if they could be established at greater than 95% probability as specified by the Peptide Prophet algorithm [91]. Protein probabilities were assigned by the Protein Prophet algorithm [90]. Protein identifications were accepted, if they reached greater than 99% probability and contained at least 2 identified unique peptides. These identification criteria typically established <0.01% FDR based on a decoy database search strategy at the protein level. Proteins that contained similar peptides and could not be differentiated based on MS/MS analysis alone were grouped to satisfy the principles of parsimony. Contaminants, and reverse identification were excluded from further data analysis.

### Semiquantitative label-free spectral counting

Significant spectral count normalized (Nsc) and fold change ratios (Rsc) were determined as previously described [70]. The relative abundance of a protein within a sample was estimated using Nsc, where for each individual protein, significant peptide MS/MS spectra (i.e., ion score greater than identity score) were summated, and normalized by the total number of significant MS/MS spectra identified in the sample. To compare relative protein abundance between samples the ratio of normalized spectral counts (Rsc) was estimated. Total number of spectra was only counted for significant peptides identified (Ion score ≥ Homology score). When Rsc is less than 1, the negative inverse value was used. For each protein the Fisher's exact test was applied to significant assigned spectra. The resulting p-values were corrected for multiple testing using the Benjamini-Hochberg procedure.

### Tumorigenicity assays

All animal experiments were approved by the Animal Care and Use Committees of the CSIC and UAM. Mice were cared for following institutional guidelines for animal care and in accordance with the standards established in the National Institutes of Health Guide for the Care and Use of Laboratory Animals. For tumorigenicity assays, ~2.4 × 10^6^ cells were intradermally injected into the two flanks of 8-10 weeks-old female Balb/c athymic nude mice (Harlan). The size of tumors was calculated from caliper measurements of two orthogonal diameters at different times. The latency of tumors was estimated as the time needed for tumors to reach a size of 0.5 cm^2^.

### *In vitro* angiogenesis and lymphangiogenesis assays

*In vitro* formation of capillary-like structures was performed on growth factor-reduced Matrigel (Corning) with primary human umbilical vein endothelial cells (HUVECs) and human dermal lymphatic microvascular endothelial cells (HLECs) purchased from Lonza. HUVECs (10^4^ cells per well) or HLECs (10^4^ or 1.2 × 10^4^ cells per well) were seeded onto Matrigel-coated wells in EBM2 (Endothelial Basic Medium) supplemented with EGM2 (Endothelial Cell Growth Medium Bulletkits™, Lonza) with or without 40 μg/ml of crude EXOs from MDCK-CMV or MDCK–PDPN. The formation of capillary-like tubular structures was recorded microscopically at the indicated times. In order to test the involvement of PDPN in PDPN-EXO stimulation of lymphangiogenesis, both types of EXOs were preincubated for 1 h at 4°C with 0.5 and 1 μg/ml of rat mAb NZ1 directed against the extracellular domain of PDPN, or with 1 μg/ml rat IgG, as a control.

### Statistical analysis

For quantification of MVs and EXOs isolated from cell lines, three independent experiments were carried out for each experimental condition. Data are presented as mean ± s.e.m. For *in vitro* angiogenesis and lymphangiogenesis assays, data are expressed as the mean ± s.e.m. of tube lengths (Image J software) in arbitrary units and/or the number of closed tube structures per field. Three different fields per well were counted in triplicate determinations. The figures shown are representative of two or three independent experiments, as indicated. Significance was determined using one-way analysis of variance followed by Bonferroni's multiple comparison test. Data were considered as significant if *p* < 0.05 was reached. All statistical analyses were performed using GraphPad Prism 5.0 software.

## SUPPLEMENTARY FIGURES AND TABLES















## References

[1] Astarita JL, Acton SE, Turley SJ (2012). Podoplanin: emerging functions in development, the immune system, and cancer. Front Immunol.

[2] Renart J, Carrasco-Ramirez P, Fernandez-Munoz B, Martin-Villar E, Montero L, Yurrita MM, Quintanilla M (2015). New insights into the role of podoplanin in epithelial-mesenchymal transition. Int Rev Cell Mol Biol.

[3] Martin-Villar E, Scholl FG, Gamallo C, Yurrita MM, Munoz-Guerra M, Cruces J, Quintanilla M (2005). Characterization of human PA2.26 antigen (T1alpha-2, podoplanin), a small membrane mucin induced in oral squamous cell carcinomas. Int J Cancer.

[4] Wicki A, Lehembre F, Wick N, Hantusch B, Kerjaschki D, Christofori G (2006). Tumor invasion in the absence of epithelial-mesenchymal transition: podoplanin-mediated remodeling of the actin cytoskeleton. Cancer Cell.

[5] Yuan P, Temam S, El-Naggar A, Zhou X, Liu DD, Lee JJ, Mao L (2006). Overexpression of podoplanin in oral cancer and its association with poor clinical outcome. Cancer.

[6] Ernst A, Hofmann S, Ahmadi R, Becker N, Korshunov A, Engel F, Hartmann C, Felsberg J, Sabel M, Peterziel H, Durchdewald M, Hess J, Barbus S, Campos B, Starzinski-Powitz A, Unterberg A (2009). Genomic and expression profiling of glioblastoma stem cell-like spheroid cultures identifies novel tumor-relevant genes associated with survival. Clin Cancer Res.

[7] Huber GF, Fritzsche FR, Zullig L, Storz M, Graf N, Haerle SK, Jochum W, Stoeckli SJ, Moch H (2011). Podoplanin expression correlates with sentinel lymph node metastasis in early squamous cell carcinomas of the oral cavity and oropharynx. Int J Cancer.

[8] Toll A, Gimeno-Beltran J, Ferrandiz-Pulido C, Masferrer E, Yebenes M, Jucgla A, Abal L, Marti RM, Sanmartin O, Baro T, Casado B, Gandarillas A, Barranco C, Costa I, Mojal S, Garcia-Patos V (2012). D2-40 immunohistochemical overexpression in cutaneous squamous cell carcinomas: a marker of metastatic risk. J Am Acad Dermatol.

[9] Atsumi N, Ishii G, Kojima M, Sanada M, Fujii S, Ochiai A (2008). Podoplanin, a novel marker of tumor-initiating cells in human squamous cell carcinoma A431. Biochem Biophys Res Commun.

[10] Kolenda J, Jensen SS, Aaberg-Jessen C, Christensen K, Andersen C, Brunner N, Kristensen BW (2011). Effects of hypoxia on expression of a panel of stem cell and chemoresistance markers in glioblastoma-derived spheroids. J Neurooncol.

[11] Bortolomai I, Canevari S, Facetti I, De Cecco L, Castellano G, Zacchetti A, Alison MR, Miotti S (2010). Tumor initiating cells: development and critical characterization of a model derived from the A431 carcinoma cell line forming spheres in suspension. Cell Cycle.

[12] Hou TZ, Bystrom J, Sherlock JP, Qureshi O, Parnell SM, Anderson G, Gilroy DW, Buckley CD (2010). A distinct subset of podoplanin (gp38) expressing F4/80+ macrophages mediate phagocytosis and are induced following zymosan peritonitis. FEBS Lett.

[13] Kerrigan AM, Navarro-Nunez L, Pyz E, Finney BA, Willment JA, Watson SP, Brown GD (2012). Podoplanin-expressing inflammatory macrophages activate murine platelets via CLEC-2. J Thromb Haemost.

[14] Yamanashi T, Nakanishi Y, Fujii G, Akishima-Fukasawa Y, Moriya Y, Kanai Y, Watanabe M, Hirohashi S (2009). Podoplanin expression identified in stromal fibroblasts as a favorable prognostic marker in patients with colorectal carcinoma. Oncology.

[15] Hoshino A, Ishii G, Ito T, Aoyagi K, Ohtaki Y, Nagai K, Sasaki H, Ochiai A (2011). Podoplanin-positive fibroblasts enhance lung adenocarcinoma tumor formation: podoplanin in fibroblast functions for tumor progression. Cancer Res.

[16] Choi SY, Sung R, Lee SJ, Lee TG, Kim N, Yoon SM, Lee EJ, Chae HB, Youn SJ, Park SM (2013). Podoplanin, alpha-smooth muscle actin or S100A4 expressing cancer-associated fibroblasts are associated with different prognosis in colorectal cancers. J Korean Med Sci.

[17] Pula B, Witkiewicz W, Dziegiel P, Podhorska-Okolow M (2013). Significance of podoplanin expression in cancer-associated fibroblasts: a comprehensive review. Int J Oncol.

[18] Yoshida T, Ishii G, Goto K, Neri S, Hashimoto H, Yoh K, Niho S, Umemura S, Matsumoto S, Ohmatsu H, Iida S, Niimi A, Nagai K, Ohe Y, Ochiai A (2015). Podoplanin-positive cancer-associated fibroblasts in the tumor microenvironment induce primary resistance to EGFR-TKIs in lung adenocarcinoma with EGFR mutation. Clin Cancer Res.

[19] Takahashi A, Ishii G, Neri S, Yoshida T, Hashimoto H, Suzuki S, Umemura S, Matsumoto S, Yoh K, Niho S, Goto K, Ohmatsu H, Nagai K, Gemma A, Ohe Y, Ochiai A (2015). Podoplanin-expressing cancer-associated fibroblasts inhibit small cell lung cancer growth. Oncotarget.

[20] Scholl FG, Gamallo C, Vilaro S, Quintanilla M (1999). Identification of PA2.26 antigen as a novel cell-surface mucin-type glycoprotein that induces plasma membrane extensions and increased motility in keratinocytes. J Cell Sci.

[21] Scholl FG, Gamallo C, Quintanilla M (2000). Ectopic expression of PA2.26 antigen in epidermal keratinocytes leads to destabilization of adherens junctions and malignant progression. Lab Invest.

[22] Martin-Villar E, Megias D, Castel S, Yurrita MM, Vilaro S, Quintanilla M (2006). Podoplanin binds ERM proteins to activate RhoA and promote epithelial-mesenchymal transition. J Cell Sci.

[23] Wicki A, Christofori G (2007). The potential role of podoplanin in tumour invasion. Br J Cancer.

[24] Nakazawa Y, Takagi S, Sato S, Oh-hara T, Koike S, Takami M, Arai H, Fujita N (2011). Prevention of hematogenous metastasis by neutralizing mice and its chimeric anti-Aggrus/podoplanin antibodies. Cancer Sci.

[25] Ochoa-Alvarez JA, Krishnan H, Shen Y, Acharya NK, Han M, McNulty DE, Hasegawa H, Hyodo T, Senga T, Geng JG, Kosciuk M, Shin SS, Goydos JS, Temiakov D, Nagele RG, Goldberg GS (2012). Plant lectin can target receptors containing sialic acid, exemplified by podoplanin, to inhibit transformed cell growth and migration. PLoS One.

[26] Abe S, Morita Y, Kaneko MK, Hanibuchi M, Tsujimoto Y, Goto H, Kakiuchi S, Aono Y, Huang J, Sato S, Kishuku M, Taniguchi Y, Azuma M, Kawazoe K, Sekido Y, Yano S (2013). A novel targeting therapy of malignant mesothelioma using anti-podoplanin antibody. J Immunol.

[27] Ochoa-Alvarez JA, Krishnan H, Pastorino JG, Nevel E, Kephart D, Lee JJ, Retzbach EP, Shen Y, Fatahzadeh M, Baredes S, Kalyoussef E, Honma M, Adelson ME, Kaneko MK, Kato Y, Young MA (2015). Antibody and lectin target podoplanin to inhibit oral squamous carcinoma cell migration and viability by distinct mechanisms. Oncotarget.

[28] Hwang YS, Xianglan Z, Park KK, Chung WY (2012). Functional invadopodia formation through stabilization of the PDPN transcript by IMP-3 and cancer-stromal crosstalk for PDPN expression. Carcinogenesis.

[29] Martin-Villar E, Borda-d'Agua B, Carrasco-Ramirez P, Renart J, Parsons M, Quintanilla M, Jones GE (2015). Podoplanin mediates ECM degradation by squamous carcinoma cells through control of invadopodia stability. Oncogene.

[30] Martin-Villar E, Fernandez-Munoz B, Parsons M, Yurrita MM, Megias D, Perez-Gomez E, Jones GE, Quintanilla M (2010). Podoplanin associates with CD44 to promote directional cell migration. Mol Biol Cell.

[31] Tsuneki M, Yamazaki M, Maruyama S, Cheng J, Saku T (2013). Podoplanin-esion through extracellular matrix in oral squamous cell carcinoma. Lab Invest.

[32] Kunita A, Kashima TG, Morishita Y, Fukayama M, Kato Y, Tsuruo T, Fujita N (2007). The platelet aggregation-inducing factor aggrus/podoplanin promotes pulmonary metastasis. Am J Pathol.

[33] Takagi S, Sato S, Oh-hara T, Takami M, Koike S, Mishima Y, Hatake K, Fujita N (2013). Platelets promote tumor growth and metastasis via direct interaction between Aggrus/podoplanin and CLEC-2. PLoS One.

[34] Uhrin P, Zaujec J, Breuss JM, Olcaydu D, Chrenek P, Stockinger H, Fuertbauer E, Moser M, Haiko P, Fassler R, Alitalo K, Binder BR, Kerjaschki D (2010). Novel function for blood platelets and podoplanin in developmental separation of blood and lymphatic circulation. Blood.

[35] Acton SE, Astarita JL, Malhotra D, Lukacs-Kornek V, Franz B, Hess PR, Jakus Z, Kuligowski M, Fletcher AL, Elpek KG, Bellemare-Pelletier A, Sceats L, Reynoso ED, Gonzalez SF, Graham DB, Chang J (2012). Podoplanin-rich stromal networks induce dendritic cell motility via activation of the C-type lectin receptor CLEC-2. Immunity.

[36] Herzog BH, Fu J, Wilson SJ, Hess PR, Sen A, McDaniel JM, Pan Y, Sheng M, Yago T, Silasi-Mansat R, McGee S, May F, Nieswandt B, Morris AJ, Lupu F, Coughlin SR (2013). Podoplanin maintains high endothelial venule integrity by interacting with platelet CLEC-2. Nature.

[37] Hur J, Jang JH, Oh IY, Choi JI, Yun JY, Kim J, Choi YE, Ko SB, Kang JA, Kang J, Lee SE, Lee H, Park YB, Kim HS (2014). Human podoplanin-positive monocytes and platelets enhance lymphangiogenesis through the activation of the podoplanin/CLEC-2 axis. Mol Ther.

[38] Pollitt AY, Poulter NS, Gitz E, Navarro-Nunez L, Wang YJ, Hughes CE, Thomas SG, Nieswandt B, Douglas MR, Owen DM, Jackson DG, Dustin ML, Watson SP (2014). Syk and Src family kinases regulate C-type lectin receptor 2 (CLEC-2)-mediated clustering of podoplanin and platelet adhesion to lymphatic endothelial cells. J Biol Chem.

[39] Peters A, Burkett PR, Sobel RA, Buckley CD, Watson SP, Bettelli E, Kuchroo VK (2015). Podoplanin negatively regulates CD4+ effector T cell responses. J Clin Invest.

[40] Barth K, Blasche R, Kasper M (2010). T1alpha/podoplanin shows raft-associated distribution in mouse lung alveolar epithelial E10 cells. Cell Physiol Biochem.

[41] Fernandez-Munoz B, Yurrita MM, Martin-Villar E, Carrasco-Ramirez P, Megias D, Renart J, Quintanilla M (2011). The transmembrane domain of podoplanin is required for its association with lipid rafts and the induction of epithelial-mesenchymal transition. Int J Biochem Cell Biol.

[42] Muralidharan-Chari V, Clancy JW, Sedgwick A, D'Souza-Schorey C (2010). Microvesicles: mediators of extracellular communication during cancer progression. J Cell Sci.

[43] Raposo G, Stoorvogel W (2013). Extracellular vesicles: exosomes, microvesicles, and friends. J Cell Biol.

[44] Colombo M, Raposo G, Thery C (2014). Biogenesis, secretion, and intercellular interactions of exosomes and other extracellular vesicles. Annu Rev Cell Dev Biol.

[45] Thery C, Ostrowski M, Segura E (2009). Membrane vesicles as conveyors of immune responses. Nat Rev Immunol.

[46] D'Souza-Schorey C, Clancy JW (2012). Tumor-derived microvesicles: shedding light on novel microenvironment modulators and prospective cancer biomarkers. Genes Dev.

[47] Greening DW, Gopal SK, Xu R, Simpson RJ, Chen W (2015). Exosomes and their roles in immune regulation and cancer. Semin Cell Dev Biol.

[48] Xu R, Greening DW, Rai A, Ji H, Simpson RJ (2015). Highly-purified exosomes and shed microvesicles isolated from the human colon cancer cell line LIM1863 by sequential centrifugal ultrafiltration are biochemically and functionally distinct. Methods.

[49] Harding CV, Heuser JE, Stahl PD (2013). Exosomes: looking back three decades and into the future. J Cell Biol.

[50] Cocucci E, Meldolesi J (2015). Ectosomes and exosomes: shedding the confusion between extracellular vesicles. Trends Cell Biol.

[51] Yonemura S, Hirao M, Doi Y, Takahashi N, Kondo T, Tsukita S, Tsukita S (1998). Ezrin/radixin/moesin (ERM) proteins bind to a positively charged amino acid cluster in the juxta-membrane cytoplasmic domain of CD44, CD43, and ICAM-2. J Cell Biol.

[52] Yurrita MM, Fernandez-Munoz B, Del Castillo G, Martin-Villar E, Renart J, Quintanilla M (2014). Podoplanin is a substrate of presenilin-1/gamma-secretase. Int J Biochem Cell Biol.

[53] Stoeck A, Keller S, Riedle S, Sanderson MP, Runz S, Le Naour F, Gutwein P, Ludwig A, Rubinstein E, Altevogt P (2006). A role for exosomes in the constitutive and stimulus-induced ectodomain cleavage of L1 and CD44. Biochem J.

[54] Bernhard OK, Greening DW, Barnes TW, Ji H, Simpson RJ (2013). Detection of cadherin-17 in human colon cancer LIM1215 cell secretome and tumour xenograft-derived interstitial fluid and plasma. Biochim Biophys Acta.

[55] Valadi H, Ekstrom K, Bossios A, Sjostrand M, Lee JJ, Lotvall JO (2007). Exosome-mediated transfer of mRNAs and microRNAs is a novel mechanism of genetic exchange between cells. Nat Cell Biol.

[56] Bolukbasi MF, Mizrak A, Ozdener GB, Madlener S, Strobel T, Erkan EP, Fan JB, Breakefield XO, Saydam O (2012). miR-1289 and “Zipcode”-like Sequence Enrich mRNAs in Microvesicles. Mol Ther Nucleic Acids.

[57] Peinado H, Aleckovic M, Lavotshkin S, Matei I, Costa-Silva B, Moreno-Bueno G, Hergueta-Redondo M, Williams C, Garcia-Santos G, Ghajar C, Nitadori-Hoshino A, Hoffman C, Badal K, Garcia BA, Callahan MK, Yuan J (2012). Melanoma exosomes educate bone marrow progenitor cells toward a pro-metastatic phenotype through MET. Nat Med.

[58] Takamatsu H, Takegahara N, Nakagawa Y, Tomura M, Taniguchi M, Friedel RH, Rayburn H, Tessier-Lavigne M, Yoshida Y, Okuno T, Mizui M, Kang S, Nojima S, Tsujimura T, Nakatsuji Y, Katayama I (2010). Semaphorins guide the entry of dendritic cells into the lymphatics by activating myosin II. Nat Immunol.

[59] Cagnoni G, Tamagnone L (2014). Semaphorin receptors meet receptor tyrosine kinases on the way of tumor progression. Oncogene.

[60] Xi HQ, Wu XS, Wei B, Chen L (2012). Aberrant expression of EphA3 in gastric carcinoma: correlation with tumor angiogenesis and survival. J Gastroenterol.

[61] Simone LC, Naslavsky N, Caplan S (2014). Scratching the surface: actin' and other roles for the C-terminal Eps15 homology domain protein, EHD2. Histol Histopathol.

[62] Andreu Z, Yanez-Mo M (2014). Tetraspanins in extracellular vesicle formation and function. Front Immunol.

[63] Nakazawa Y, Sato S, Naito M, Kato Y, Mishima K, Arai H, Tsuruo T, Fujita N (2008). Tetraspanin family member CD9 inhibits Aggrus/podoplanin-induced platelet aggregation and suppresses pulmonary metastasis. Blood.

[64] Yoshioka Y, Konishi Y, Kosaka N, Katsuda T, Kato T, Ochiya T (2013). Comparative marker analysis of extracellular vesicles in different human cancer types. J Extracell Vesicles.

[65] Haqqani AS, Delaney CE, Tremblay TL, Sodja C, Sandhu JK, Stanimirovic DB (2013). Method for isolation and molecular characterization of extracellular microvesicles released from brain endothelial cells. Fluids Barriers CNS.

[66] Stenmark H (2009). Rab GTPases as coordinators of vesicle traffic. Nat Rev Mol Cell Biol.

[67] Hutagalung AH, Novick PJ (2011). Role of Rab GTPases in membrane traffic and cell physiology. Physiol Rev.

[68] Baietti MF, Zhang Z, Mortier E, Melchior A, Degeest G, Geeraerts A, Ivarsson Y, Depoortere F, Coomans C, Vermeiren E, Zimmermann P, David G (2012). Syndecan-syntenin-ALIX regulates the biogenesis of exosomes. Nat Cell Biol.

[69] Garnier D, Magnus N, Meehan B, Kislinger T, Rak J (2013). Qualitative changes in the proteome of extracellular vesicles accompanying cancer cell transition to mesenchymal state. Exp Cell Res.

[70] Tauro BJ, Mathias RA, Greening DW, Gopal SK, Ji H, Kapp EA, Coleman BM, Hill AF, Kusebauch U, Hallows JL, Shteynberg D, Moritz RL, Zhu HJ, Simpson RJ (2013). Oncogenic H-ras reprograms Madin-Darby canine kidney (MDCK) cell-derived exosomal proteins following epithelial-mesenchymal transition. Mol Cell Proteomics.

[71] Edmond V, Dufour F, Poiroux G, Shoji K, Malleter M, Fouque A, Tauzin S, Rimokh R, Sergent O, Penna A, Dupuy A, Levade T, Theret N, Micheau O, Segui B, Legembre P (2015). Downregulation of ceramide synthase-6 during epithelial-to-mesenchymal transition reduces plasma membrane fluidity and cancer cell motility. Oncogene.

[72] Mooren OL, Galletta BJ, Cooper JA (2012). Roles for actin assembly in endocytosis. Annu Rev Biochem.

[73] Hoshino D, Kirkbride KC, Costello K, Clark ES, Sinha S, Grega-Larson N, Tyska MJ, Weaver AM (2013). Exosome secretion is enhanced by invadopodia and drives invasive behavior. Cell Rep.

[74] Breiteneder-Geleff S, Soleiman A, Kowalski H, Horvat R, Amann G, Kriehuber E, Diem K, Weninger W, Tschachler E, Alitalo K, Kerjaschki D (1999). Angiosarcomas express mixed endothelial phenotypes of blood and lymphatic capillaries: podoplanin as a specific marker for lymphatic endothelium. Am J Pathol.

[75] Navarro A, Perez RE, Rezaiekhaligh M, Mabry SM, Ekekezie II (2008). T1alpha/podoplanin is essential for capillary morphogenesis in lymphatic endothelial cells. Am J Physiol Lung Cell Mol Physiol.

[76] Navarro A, Perez RE, Rezaiekhaligh MH, Mabry SM, Ekekezie II (2011). Polarized migration of lymphatic endothelial cells is critically dependent on podoplanin regulation of Cdc42. Am J Physiol Lung Cell Mol Physiol.

[77] Cueni LN, Chen L, Zhang H, Marino D, Huggenberger R, Alitalo A, Bianchi R, Detmar M (2010). Podoplanin-Fc reduces lymphatic vessel formation in vitro and in vivo and causes disseminated intravascular coagulation when transgenically expressed in the skin. Blood.

[78] Kerjaschki D, Regele HM, Moosberger I, Nagy-Bojarski K, Watschinger B, Soleiman A, Birner P, Krieger S, Hovorka A, Silberhumer G, Laakkonen P, Petrova T, Langer B, Raab I (2004). Lymphatic neoangiogenesis in human kidney transplants is associated with immunologically active lymphocytic infiltrates. J Am Soc Nephrol.

[79] Stacker SA, Williams SP, Karnezis T, Shayan R, Fox SB, Achen MG (2014). Lymphangiogenesis and lymphatic vessel remodelling in cancer. Nat Rev Cancer.

[80] Mathias RA, Lim JW, Ji H, Simpson RJ (2009). Isolation of extracellular membranous vesicles for proteomic analysis. Methods Mol Biol.

[81] Ehrlich M, Shmuely A, Henis YI (2001). A single internalization signal from the di-leucine family is critical for constitutive endocytosis of the type II TGF-beta receptor. J Cell Sci.

[82] Greening DW, Xu R, Ji H, Tauro BJ, Simpson RJ (2015). A protocol for exosome isolation and characterization: evaluation of ultracentrifugation, density-gradient separation, and immunoaffinity capture methods. Methods Mol Biol.

[83] Ji H, Greening DW, Barnes TW, Lim JW, Tauro BJ, Rai A, Xu R, Adda C, Mathivanan S, Zhao W, Xue Y, Xu T, Zhu HJ, Simpson RJ (2013). Proteome profiling of exosomes derived from human primary and metastatic colorectal cancer cells reveal differential expression of key metastatic factors and signal transduction components. Proteomics.

[84] Thery C, Amigorena S, Raposo G, Clayton A (2006). Isolation and characterization of exosomes from cell culture supernatants and biological fluids. Curr Protoc Cell Biol.

[85] Shevchenko A, Tomas H, Havlis J, Olsen JV, Mann M (2006). In-gel digestion for mass spectrometric characterization of proteins and proteomes. Nat Protoc.

[86] Rappsilber J, Mann M, Ishihama Y (2007). Protocol for micro-purification, enrichment, pre-fractionation and storage of peptides for proteomics using StageTips. Nat Protoc.

[87] Gopal SK, Greening DW, Mathias RA, Ji H, Rai A, Chen M, Zhu HJ, Simpson RJ (2015). YBX1/YB-1 induces partial EMT and tumourigenicity through secretion of angiogenic factors into the extracellular microenvironment. Oncotarget.

[88] Brosch M, Yu L, Hubbard T, Choudhary J (2009). Accurate and sensitive peptide identification with Mascot Percolator. J Proteome Res.

[89] Greening DW, Simpson RJ (2013). An updated secretome. Biochim Biophys Acta.

[90] Nesvizhskii AI, Aebersold R (2005). Interpretation of shotgun proteomic data: the protein inference problem. Mol Cell Proteomics.

[91] Keller A, Nesvizhskii AI, Kolker E, Aebersold R (2002). Empirical statistical model to estimate the accuracy of peptide identifications made by MS/MS and database search. Anal Chem.

