# Supplementary material for: Podoplanin is a component of extracellular vesicles that reprograms cell-derived exosomal proteins and modulates lymphatic vessel formation
Source: Oncotarget. 2016 Feb 17;7(13):16070–89. doi: 10.18632/oncotarget.7445 (PMC4941298; doi:10.18632/oncotarget.7445)
Supplement: Supplementary file 1 [file oncotarget-07-16070-s001.pdf]

SUPPLEMENTARY INFORMATION

Cellular proteome analysis of MDCK-CMV and MDCK-PDPN

Many proteins highly upregulated in MDCK-PDPN cells (Supplementary Table 1) are overexpressed in different types of tumors and promote tumorigenesis and/or metastasis. This is the case of SERPINE1/PAI-1, which promotes cancer cell proliferation, survival and migration through modulating the function of urokinase-type plasminogen activator receptor [1], and also was found to be upregulated in MDCK cells upon expression of EMT-associated transcription factors Snail1, Snail2 or E47 [2]. Likewise, PLOD2, a key mediator of collagen cross-linking that increases fibrillar collagen formation and tumor stiffness, favoring local and distal metastases [3], and PTPN1/PTP1B, a key regulator of signaling networks implicated in metabolic diseases, which stimulates tumor progression [4]. Cancer cells undergo the Warburg effect characterized by a high rate of glycolysis and acid lactic fermentation, even under normoxic conditions, allowing them to be glutamine addicted. The key enzyme for glutamine addiction is glutaminase (GLS), which is upregulated in MDCK-PDPN cells as well as in different types of tumors [5]. Overexpression of GLS allows MDCK-PDPN cells a typical trait of neoplastic cells.

In addition, most cellular proteins downregulated in MDCK-PDPN (Supplementary Table 2) act as tumor suppressors in different types of malignancies. Particularly interesting, in this respect, are AP1B1 and LLGL2, both of which are involved in the maintenance of epithelial cell polarity by regulating apical and basolateral intracellular trafficking [6, 7]. Disrupted cell polarity is a hallmark of epithelial cancers [8]. For instance, LLGL2 is a tumor suppressor whose downregulation in gastric and endometrial cancers is associated with EMT [7]. Among proteins whose expression diminishes in MDCK-PDPN cells, it is worth emphasizing the downregulation of several key proteins: ESPR1 is related to the global switch in splicing associated with EMT [9], and, likely, is in the origin of the switch in splice variants observed in p120 catenin and CD44 during PDPN-induced EMT [10, 11]; SERPINB5 is a non-inhibitory member of the serpin family that, contrarily to SERPINE1/PAI-1, inhibits cancer cell migration [1]; while ISG15 is an antagonist of the canonical ubiquitin pathway that is found conjugated to cellular proteins (ISGylation) during cancer. ISGylation has a pro-tumorigenic function, but free extracellular ISG15 acts as an immunomodulatory cytokine and is a tumor suppressor [12]. It is interesting to note that downregulation of ISG15 in MDCK-PDPN cells might be related to this latter property.

REFERENCES

1. Zheng D, Chen H, Davids J, Bryant M and Lucas A. Serpins for diagnosis and therapy in cancer. Cardiovasc Hematol Disord Drug Targets. 2013; 13:123-132.
2. Moreno-Bueno G, Peinado H, Molina P, Olmeda D, Cubillo E, Santos V, Palacios J, Portillo F and Cano A. The morphological and molecular features of the epithelial-to-mesenchymal transition. Nat Protoc. 2009; 4:1591-1613.
3. Chen Y, Terajima M, Yang Y, Sun L, Ahn YH, Pankova D, Puperi DS, Watanabe T, Kim MP, Blackmon SH, Rodriguez J, Liu H, Behrens C, Wistuba, II, Minelli R, Scott KL, et al. Lysyl hydroxylase 2 induces a collagen cross-link switch in tumor stroma. J Clin Invest. 2015; 125:1147-1162.
4. Bakke J and Haj FG. Protein-tyrosine phosphatase 1B substrates and metabolic regulation. Semin Cell Dev Biol. 2015; 37:58-65.
5. Katt WP and Cerione RA. Glutaminase regulation in cancer cells: a druggable chain of events. Drug Discov Today. 2014; 19:450-457.
6. Folsch H. The building blocks for basolateral vesicles in polarized epithelial cells. Trends Cell Biol. 15:222-228.
7. Halaoui R and McCaffrey L. Rewiring cell polarity signaling in cancer. Oncogene. 2015; 34:939-950.
8. Martin-Belmonte F and Perez-Moreno M. Epithelial cell polarity, stem cells and cancer. Nat Rev Cancer. 2012; 12:23-38.
9. Warzecha CC and Carstens RP. Complex changes in alternative pre-mRNA splicing play a central role in the epithelial-to-mesenchymal transition (EMT). Semin Cancer Biol. 2012; 22:417-427.
10. Martin-Villar E, Megias D, Castel S, Yurrita MM, Vilaro S and Quintanilla M. Podoplanin binds ERM proteins to activate RhoA and promote epithelial-mesenchymal transition. J Cell Sci. 2006; 119:4541-4553.
11. Martin-Villar E, Fernandez-Munoz B, Parsons M, Yurrita MM, Megias D, Perez-Gomez E, Jones GE and Quintanilla M. Podoplanin associates with CD44 to promote directional cell migration. Mol Biol Cell. 2010; 21:4387-4399.
12. Burks J, Reed RE and Desai SD. Free ISG15 triggers an antitumor immune response against breast cancer: a new perspective. Oncotarget. 2015; 6:7221-7231.
13. Bolukbasi MF, Mizrak A, Ozdener GB, Madlener S, Strobel T, Erkan EP, Fan JB, Breakefield XO and Saydam O. miR-1289 and "Zipcode"-like Sequence Enrich mRNAs in Microvesicles. Mol Ther Nucleic Acids. 2012; 1:e10.

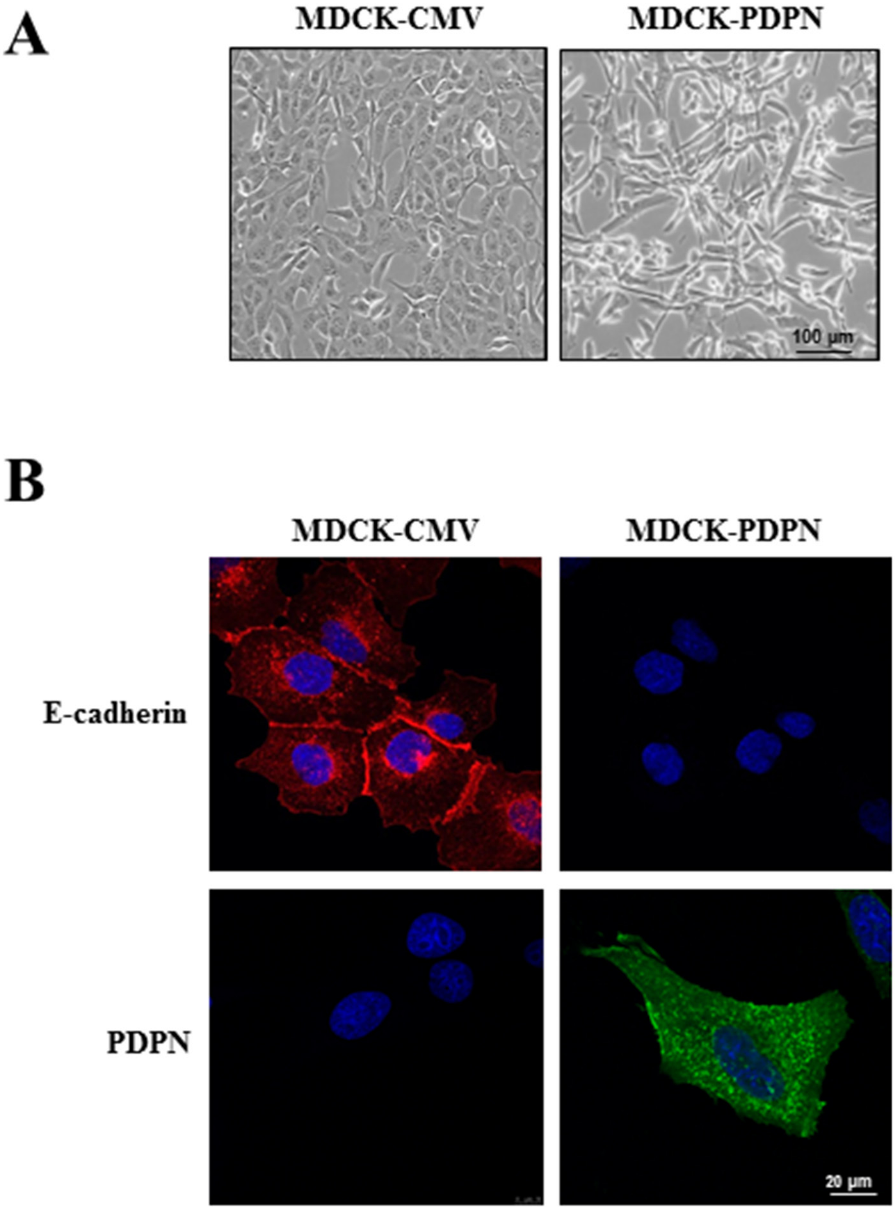

Figure S1: PDPN expression induces an EMT in MDCK cells. A. Phase contrast micrographs of MDCK-CMV and MDCK-PDPN cells showing epithelial and fibroblast-like morphologies, respectively. Scale bar, 100 µm **B.** Immunofluorescence localization of E-cadherin and PDPN. Note that E-cadherin is downregulated in cells expressing PDPN. Immunofluorescence analysis was performed as described in [10]. Scale bar, 20 µm.

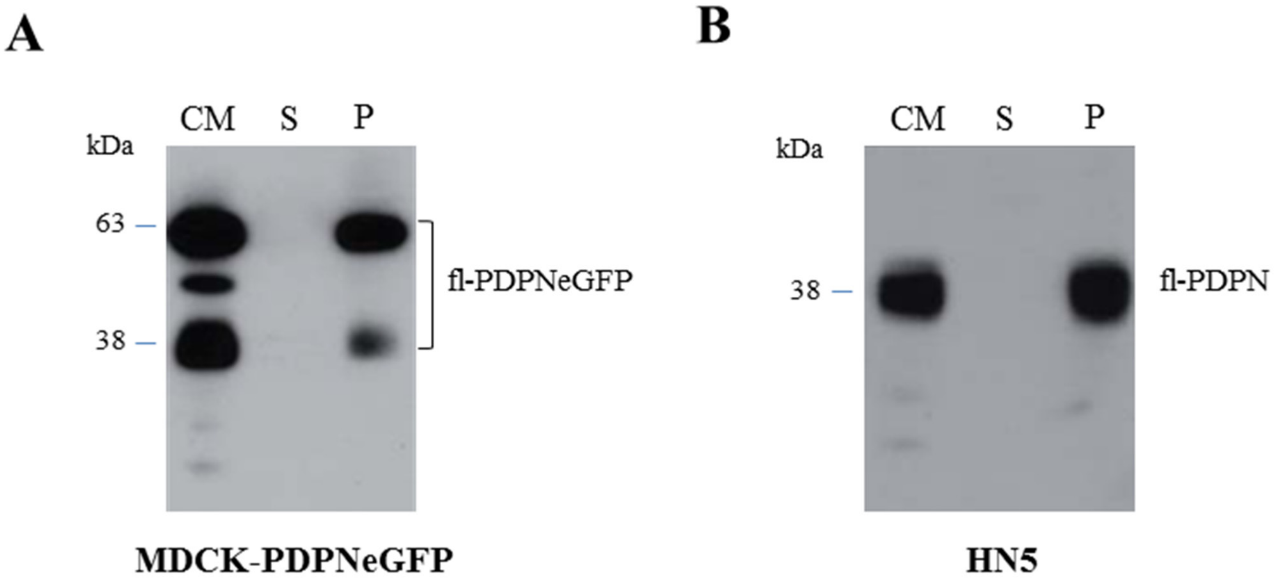

Figure S2: Western blots showing PDPN detection in the conditioned medium (CM) and pellet (P) fraction of MDCK-PDPNeGFP A. and HN5 B. cells. Cells were conditioned with serum-free medium for 24 h. The P fraction was obtained after ultracentrifugation (100,000 x *g*, 1 h) of the CM. S, supernatant. Note that the full-length (fl) protein is detected in the CM and P fraction of both cell lines.

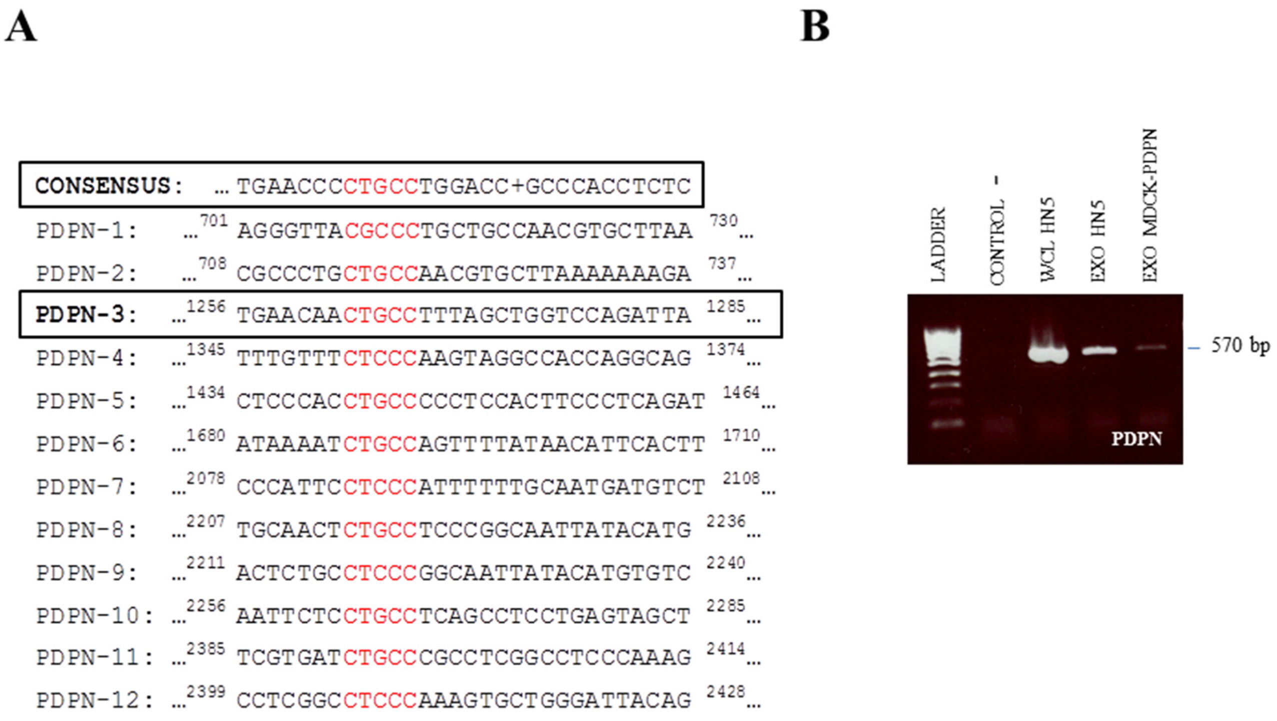

Figure S3: EVs incorporate PDPN mRNA. A. Alignment of 3'UTR sequences of PDPN mRNA with "zipcode" consensus sequence of transcripts enriched in EVs [13]. The 5-nt core sequence is shown in red. The sequence with the highest correspondence appears in the rectangle. **B.** Analysis of PDPN and CD44 transcript expression in HN5 and MDCK-PDPN cells and EVs by RT-PCR.

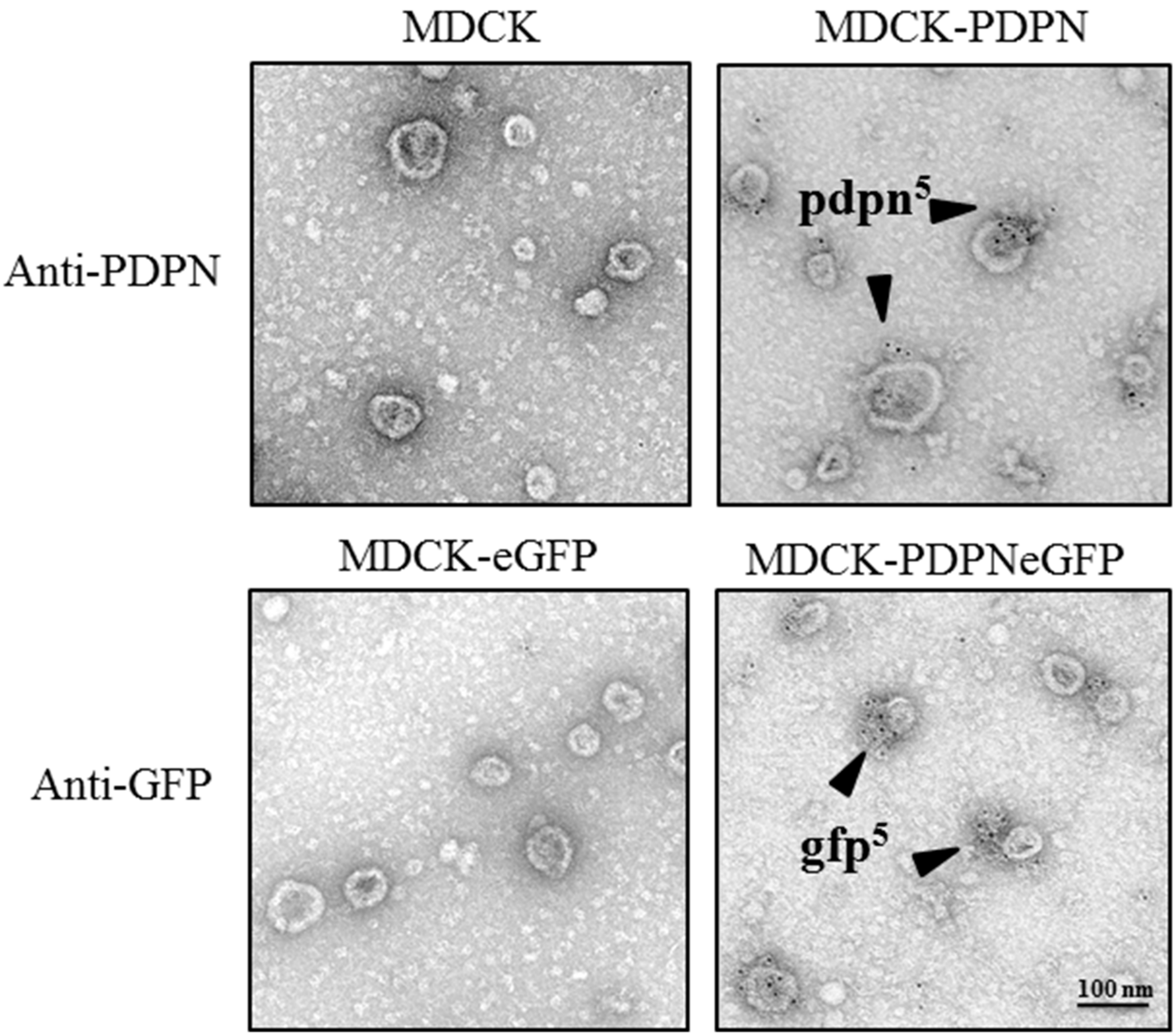

Figure S4: PDPN immunoelectron microscopy of EVs isolated from MDCK-PDPN and MDCK-PDPNeGFP cells. Untagged PDPN and PDPNeGFP were detected with specific polyclonal Abs directed either against the extracellular domain of PDPN (pdpn) and the intracellular tag (gfp), respectively, and protein A conjugated to 5-nm gold particles. Scale bar, 100 nm.

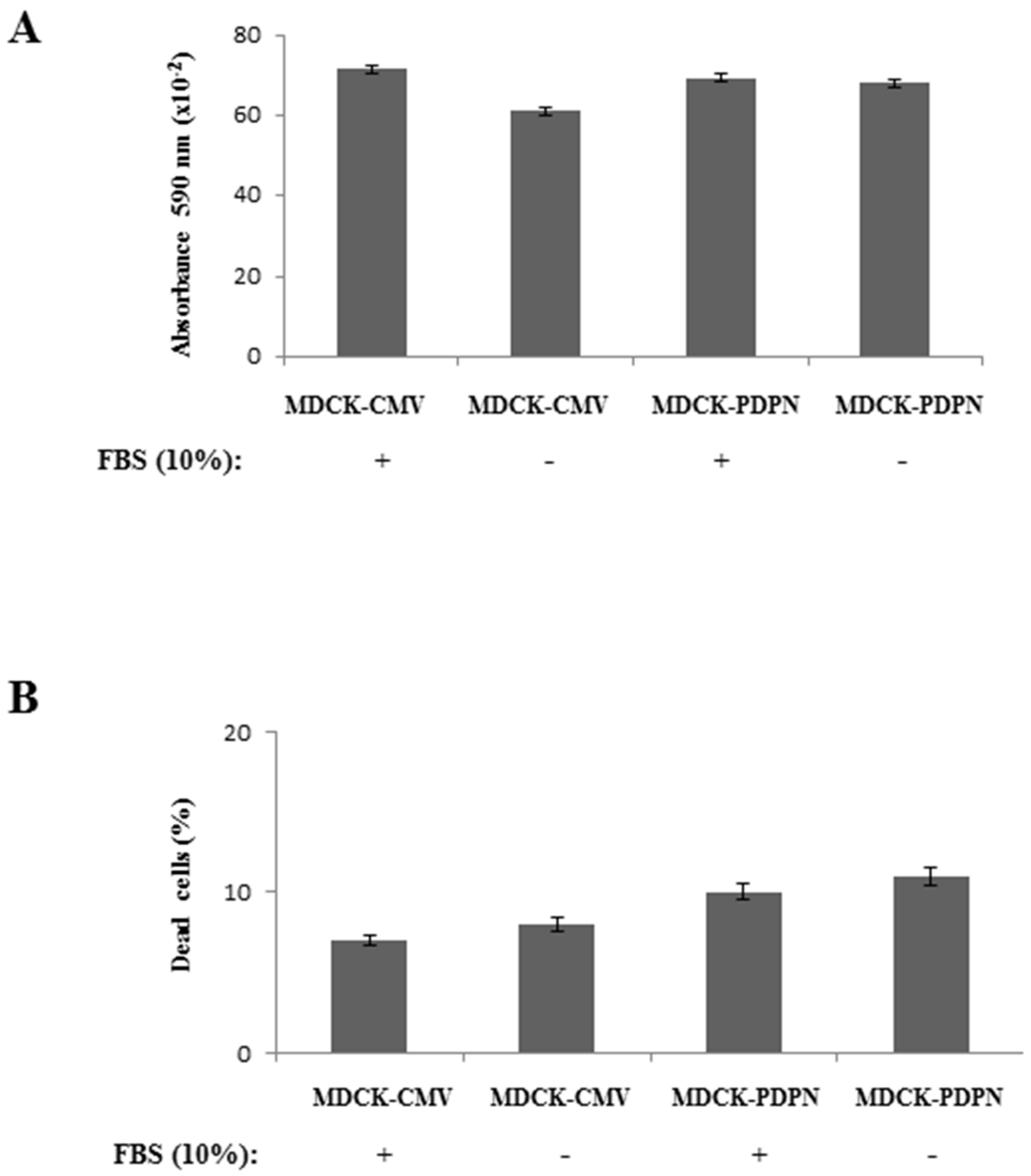

Figure S5: Culture conditions in the absence of FBS do not affect cell proliferation and viability. MDCK-CMV and MDCK-PDPN cells were cultured in the absence or presence of 10% FBS for 24 h, and cell growth **A.** and viability **B.** were determined by the MTT assay and trypan blue dye-exclusion, respectively.

Supplementary Table S1: List of proteins upregulated by more than 5 fold in MDCK-PDPN *vs* MDCK-CMV cells

See Supplementary File 1

Supplementary Table S2: List of proteins downregulated by more than 5 fold in MDCK-PDPN *vs* MDCK-CMV cells

See Supplementary File 2

Supplementary Table S3: Proteins involved in the control of endocytosis and vesicle trafficking enriched in MDCK-PDPN *vs* MDCK-CMV EXOs

See Supplementary File 3

Supplementary Table S4: Cell adhesion and cytoskeletal proteins enriched in MDCK-PDPN *vs* MDCK-CMV EXOs

See Supplementary File 4

Supplementary Table S5: Components of signal transduction pathways enriched in MDCK-PDPN *vs* MDCK-CMV EXOs

See Supplementary File 5

Supplementary Table S6: Epithelial protein markers decreased in MDCK-PDPN *vs* MDCK-CMV EXOs

See Supplementary File 6
